# A Scalable Risk-Scoring System Based on Consumer-Grade Wearables for Inpatients With COVID-19: Statistical Analysis and Model Development

**DOI:** 10.2196/35717

**Published:** 2022-06-21

**Authors:** Simon Föll, Adrian Lison, Martin Maritsch, Karsten Klingberg, Vera Lehmann, Thomas Züger, David Srivastava, Sabrina Jegerlehner, Stefan Feuerriegel, Elgar Fleisch, Aristomenis Exadaktylos, Felix Wortmann

**Affiliations:** 1 Department of Management, Technology, and Economics ETH Zürich Zürich Switzerland; 2 Department of Emergency Medicine Inselspital, Bern, University Hospital University of Bern Bern Switzerland; 3 Department of Diabetes, Endocrinology, Nutritional Medicine and Metabolism Inselspital, Bern, University Hospital University of Bern Bern Switzerland; 4 Department of Endocrinology, Diabetes and Metabolic Diseases Kantonsspital Olten Olten Switzerland; 5 Institute of AI in Management LMU Munich Munich Germany; 6 Institute of Technology Management University of St. Gallen St. Gallen Switzerland

**Keywords:** COVID-19, risk scoring, wearable devices, wearable, smartwatches, smartwatch, Bayesian survival analysis, remote monitoring, patient monitoring, remote patient monitoring, smart device, digital health, risk score, scalable, general ward, hospital, measurement tool, measurement instrument

## Abstract

**Background:**

To provide effective care for inpatients with COVID-19, clinical practitioners need systems that monitor patient health and subsequently allow for risk scoring. Existing approaches for risk scoring in patients with COVID-19 focus primarily on intensive care units (ICUs) with specialized medical measurement devices but not on hospital general wards.

**Objective:**

In this paper, we aim to develop a risk score for inpatients with COVID-19 in general wards based on consumer-grade wearables (smartwatches).

**Methods:**

Patients wore consumer-grade wearables to record physiological measurements, such as the heart rate (HR), heart rate variability (HRV), and respiration frequency (RF). Based on Bayesian survival analysis, we validated the association between these measurements and patient outcomes (ie, discharge or ICU admission). To build our risk score, we generated a low-dimensional representation of the physiological features. Subsequently, a pooled ordinal regression with time-dependent covariates inferred the probability of either hospital discharge or ICU admission. We evaluated the predictive performance of our developed system for risk scoring in a single-center, prospective study based on 40 inpatients with COVID-19 in a general ward of a tertiary referral center in Switzerland.

**Results:**

First, Bayesian survival analysis showed that physiological measurements from consumer-grade wearables are significantly associated with patient outcomes (ie, discharge or ICU admission). Second, our risk score achieved a time-dependent area under the receiver operating characteristic curve (AUROC) of 0.73-0.90 based on leave-one-subject-out cross-validation.

**Conclusions:**

Our results demonstrate the effectiveness of consumer-grade wearables for risk scoring in inpatients with COVID-19. Due to their low cost and ease of use, consumer-grade wearables could enable a scalable monitoring system.

**Trial Registration:**

Clinicaltrials.gov NCT04357834; https://www.clinicaltrials.gov/ct2/show/NCT04357834

## Introduction

Health trajectories from patients with COVID-19 show large variability with sudden deterioration in the disease state and uncertain outcomes [1-4]. Hence, to provide effective care, clinical practitioners need systems that allow for monitoring the health trajectory of patients with COVID-19, especially during hospitalization [5-8]. Such systems can then be used to estimate the risk of a deterioration in the health condition and thus generate early warnings of critical conditions. In clinical practice, this enables the allocation of resources to patients in need and supports early responses to critical conditions [6,9-11].

Prior research has developed systems for monitoring patients with COVID-19 in different settings. One research stream detects the onset of COVID-19 using wearables (eg, smartphones) [12-14] and thus addresses the time before hospitalization. Another literature stream focuses on risk scoring for patients in intensive care units (ICUs) [7,8,15-17]. Here, monitoring systems are customized for the needs in intensive care and thus build upon specialized and often proprietary medical devices for physiological measurements. Vital signs, such as the heart rate (HR) or respiration frequency (RF), have been found to be predictive of critical health conditions [7,16]. In contrast, research is needed that develops systems for risk scoring for inpatients in general wards, which presents the focus of this work. This requires a custom risk score tailored to the corresponding patient population and nonspecialized monitoring devices that are available in general wards. For inpatients with COVID-19 in general wards, we propose the use of consumer-grade wearables (smartwatches) for monitoring and subsequent risk scoring due to their low cost, ease of use, and, thus, potential scalability. Previously, research has demonstrated the clinical relevance of consumer-grade wearables for longitudinal physiological measurements [18,19]. Further, they have been used for monitoring the progression of various other diseases (eg, diabetes mellitus [20,21]), yet their effectiveness for inpatients with COVID-19 in general wards remains to be confirmed.

**Figure 1:**
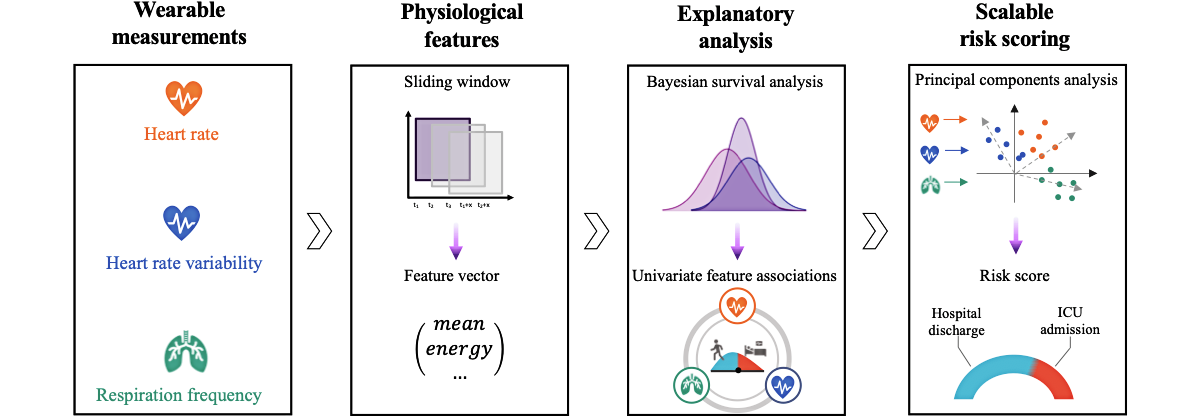
Monitoring and risk scoring. To develop a scalable risk score, physiological features were computed from wearable measurements. Next, Bayesian survival analysis was conducted to assess the association between the physiological features and patient outcomes. Lastly, a scalable risk score was developed. This study was designed to demonstrate the effectiveness of consumer-grade wearables for a scalable risk-scoring system in inpatients with COVID-19 in the general ward. ICU: intensive care unit.

In this paper, we develop a risk score for inpatients with COVID-19 in general wards based on scalable consumer-grade wearables (see Figure 1 for our overview). The consumer-grade wearables are used to monitor physiological measurements of patients: HR, heart rate variability (HRV), and RF. Based on these measurements, our risk score assesses the risk of different patient outcomes, defined as the probability of hospital discharge and ICU admission.

## Methods

### Study Procedure

In visit 1 (V1), a study investigator explained the nature, purpose, and risks of the study and provided eligible patients with a copy of the patient information sheet. If written informed consent was obtained and eligibility criteria were met, the remaining screening information was obtained. A patient number was assigned to each patient in ascending order.

Eligible patients were provided with a Garmin vívoactive 4S (Garmin International Inc., Olathe, Kansas, USA) smartwatch and a Xiaomi Redmi 9 (Xiaomi Corp., Beijing, China) smartphone. Patients wore the wearable on the wrist of the dominant hand, if possible (otherwise the other hand).

After mounting the devices, the study investigator controlled the function of the devices and checked whether data transfer was working properly. In addition, the patient was instructed to fully charge both devices once per day or as needed. The smartwatch was worn during the whole study duration, that is, from hospitalization in the general ward until the patient was admitted to the ICU or discharged home.

The study investigators were equipped with a monitoring dashboard allowing for observation of the charging status as well as functionality of the devices in use. If a patient was not capable of charging the devices themselves or the devices were not working properly, a member of the study team directly approached the patient and either charged the devices or solved possible technical issues.

In visit 2 (V2, close-out visit), the treating physician in the general ward informed the study team that 1 of the close-out criteria (admitted to the ICU or discharged home) had been met. A member of the study team then visited the patient and initiated the close-out visit. During V2, patients returned the wearable, the smartphone, and the charging cable. Completeness of data transfer to the back-end server was checked, and thereafter, all data on the devices were deleted.

### Ethical Considerations

The study followed the Declaration of Helsinki, the guidelines of good clinical practice, Swiss health laws, and the ordinance on clinical research. The study was approved by the local ethics committee of Bern, Switzerland (ID 2020-00874). Each patient provided informed written consent before any study-related procedure.

### Data Collection

The technical backbone of our data collection comprised 2 components: (1) a smartwatch that continuously collected physiological parameters and (2) a custom smartphone app to transfer the data to our server. In particular, the collected data were first transferred via Bluetooth to our self-developed smartphone app. Subsequently, the data were sent to a central database.

The smartwatch was used for measuring various physiological parameters. The recorded sensor measurements were the accelerometer (ACC), interbeat interval (IBI), HR, and RF. The ACC was sampled with 25 Hz. The HR and RF were logged once per minute. The IBI was recorded by logging the time of each heartbeat. The HR, IBI, and RF were derived from the photoplethysmography (PPG) sensor of the wearable.

Additional patient demographics (ie, patient age and sex) were collected by the clinical practitioners.

### Data Processing

Data gathered from sensors embedded in consumer-grade wearable devices come along with inherent challenges for clinical usage. In particular, consumer smartwatches are by no means certified medical devices, and their sensor data may be subject to noise and missing values. We thus performed customized preprocessing of the sensor data as follows.

The HRV was computed based on a time series of IBIs. Of note, variability measures retrieved from an optical PPG signal should be referred to as pulse rate variability (PRV), whereas the variability measures retrieved from an electrocardiogram (ECG) should be referred to as the HRV. Since variability measures are significantly correlated, we followed the convention and speak here of the HRV [22-24]. First, measurement artifacts were filtered by removing IBIs that differed by more than 20% from the preceding IBI [25]. Furthermore, we used an adaptive threshold analysis for the HRV that discarded time windows with less than half of the expected heartbeats recorded by the measurement device [26]. This adaptive threshold prevents HRV values from being distorted due to insufficient data in a time window. Subsequently, the HRV in both the time domain and the frequency domain was calculated according to international guidelines [25]. For the frequency-domain features, one needs to estimate the power spectral density [27]. The time between 2 heartbeats changes. Hence, the IBI series is irregularly sampled. To avoid resampling, which bears the risk of distorted HRV features in case the proportion of missing data increases, we relied on the Lomb-Scargle method [28-32].

### Feature Engineering

Our data showed substantial variation in the HR, HRV, and RF throughout the day, which was most likely due to changes in patient activity patterns. A confirmatory check showed strong dependence on the intensity of body movements throughout the day (see Multimedia Appendix 1). To compute daily physiological features that are robust against the activity patterns of patients and their biological rhythms, measurements taken during a time window from midnight to 5:00 a.m. each day were used. This time frame roughly corresponds to the phase of patients’ night rest, as characterized by stable physiological measurements and minimal body movements (see Multimedia Appendix 1).

The wearable-based measurements of the HR, HRV, and RF were aggregated into a single value per time window using feature engineering. For the HR and RF, we computed 15 statistical features that reflect different properties of the distribution over time (eg, mean, skewness, SD). For the HRV, there exists an extensive amount of research on the effect of the window size on HRV features [33-36]. Here, we followed recommendations by Malik et al [25] and computed 19 time-domain and frequency-domain HRV features over intervals of 300 seconds before taking the mean over the full time window. The detailed list of features is provided in Multimedia Appendix 1. To ensure representativeness of the features, we required a sufficient number of valid measurements during the night—reasonably, minimum data of half of the measuring period during the night. After the application of all quality criteria (ie, IBI quality criteria and minimum coverage of the measurement period), 114 (69.1%) of 165 observations were retained. Here, 1 observation represents the aggregated physiological measurements of a patient from 1 specific night. Throughout the paper, the combination of wearable-based measurements (HR, HRV, RF) and feature engineering is referred to as *physiological features*.

For both preprocessing and feature engineering, we leveraged the publicly available Python package FLIRT, which is tailored to process wearable data [37]. By choosing the parameters as stated before, the entire pipeline can be reproduced.

### Explanatory Analysis of the Association of Physiological Features With Patient Outcomes

To assess the association of physiological features with observed patient outcomes (ie, hospital discharge vs ICU admission), survival analysis was conducted. This allowed us to appropriately account for the time-to-event nature of the data and the presence of right censoring (see the Results section). Since the physiological features were updated each day, they were represented by time-dependent covariates in our survival analysis. Accordingly, a pooled regression approach [38,39] was chosen to flexibly account for the time dependence of the covariates. Moreover, as we did not observe an ICU readmission after a hospital discharge, both events should be regarded as competing risks and modeled via cause-specific hazards [40]. To make optimal use of the data in our study, both hazards for hospital discharge and ICU admission were estimated in a joint model. Specifically, the probabilities of hospital discharge, ICU admission, or no event (ie, continued stay) of patient “i” on day “t” were related to a regression function of the physiological features from the previous night. The probabilities were modeled jointly via an ordinal regression using a cumulative probability model with complementary log-log link [41-43]. This can also be interpreted as modeling the health condition of patients through a latent variable, where hospital discharge indicates a better health condition than continued stay and continued stay indicates a better health condition than ICU admission. An ordinal regression model accurately reflects this relationship, while offering high flexibility. Additionally, patient age and sex were considered demographic features. The model was specified in a fully Bayesian framework. Thereby, we ensured the robustness of our analysis by appropriately quantifying the uncertainty in parameter estimates. This is particularly important for limited sample sizes, as may likely be the case with newly emerged diseases. The formal specification of our model is provided in Multimedia Appendix 2. Of note, our approach has a particular connection with the well-known proportional hazards model [44] and can be interpreted as a Cox regression with time-dependent covariates that further accounts for competing risks in a joint model of hospital discharge and ICU admission probability.

In our explanatory analysis, we estimated univariate associations, which allowed us to identify the association of individual physiological features with patient health. Thus, a separate model was fitted for each physiological feature.

### Development of a Risk Score

To develop a risk score based on the physiological features, a parsimonious 2-step approach was chosen. That is, we first used feature engineering and principal component analysis (PCA) to obtain a low-dimensional but comprehensive representation of patients’ physiological state. This representation was then linked to patient outcomes through a Bayesian survival model that was similar to the models used in our explanatory analysis. We chose this approach over alternative methods (eg, deep learning) due to several reasons. First, the use of a parametric model can effectively reduce the risk of overfitting, while the feature engineering still allows us to use high-dimensional sensor data. Moreover, by jointly modeling the probabilities of hospital discharge and ICU admission, the risk score makes optimal use of the available data and can be readily interpreted as an overall indicator of patient condition. Finally, the use of Bayesian modeling ensures robust results even with limited amounts of data and appropriately quantifies uncertainty in the risk score.

The risk score was constructed by combining multiple physiological features into an overall metric. For this, we proceeded as follows: (1) The coefficients of the explanatory models were used to select physiological features of the HR, HRV, and RF that showed a relevant association (80% credible interval [CrI], excluding 0) with patient outcomes. (2) Since many features of the same measurement were strongly correlated, dimensionality reduction via PCA [45] was applied to generate a lower-dimensional representation of the underlying physiological information. (3) Pooled logistic least absolute shrinkage and selection operator (LASSO) regressions were employed to identify principal components (PCs) with high predictive power. Here, the PCs were used as predictors for the probability of either hospital discharge or ICU admission on a given day. The tuning parameter *λ* for the LASSO regularization was chosen via cross-validation. All PCs with nonzero coefficients were selected. (4) The risk score was computed from the linear predictor of a similar ordinal regression model as for the explanatory analysis but with the selected PCs as covariates. Correspondingly, a larger risk score implies a higher probability of ICU admission and a lower probability of hospital discharge. The probability of continued stay (ie, neither hospital discharge nor ICU admission) is P_continued stay_ = 1 – P_discharge_ – P_ICU_.

### Estimation and Performance Evaluation

All model parameters were estimated using a fully Bayesian framework [46-48]. Weakly informative priors were used for all parameters [49], and the estimation was checked by following best-practice recommendations in Bayesian modeling [46,50]. Details of the estimation and model checking are provided in Multimedia Appendices 2 and 3.

The performance of the developed risk score was evaluated via leave-one-patient-out cross-validation. The cross-validation covered all relevant preprocessing steps, including PCA. We assessed the performance in terms of discrimination accuracy via time-dependent receiver operating characteristic (ROC) curves using the incident case approach with dynamic controls (I/D) [51,52]. Moreover, in survival models with competing risks, ROC curves must be cause specific. Due to the small number of patients with ICU admission in our sample, we here focused on the ROC curve for hospital discharge. The ROC curve of the risk score–based prediction of hospital discharge for varying length of stay was thus evaluated to obtain a time-dependent area under the receiver operating characteristic curve (AUROC; see Multimedia Appendix 2 for details). The time-dependent AUROC assesses the predictive accuracy of the risk score to discriminate between patients who are discharged after a given number of days and patients who continue to stay in the hospital [52]. It can be interpreted as the probability that a random patient who is discharged on day “t” has a higher predicted hazard of discharge than a random patient who continues to stay in the hospital [53].

To assess the added value of continuous physiological measurements for monitoring a patient’s health throughout their hospital stay, we compared our risk score model to an alternative model that uses only data from the first night of hospital stay but is otherwise identical. The time-dependent AUROC was computed for both risk score models and compared across a varying length of stay in the hospital. We evaluated the length of stay for which a sufficient number of observations was available (ie, up to 6 days), corresponding to 87% of all observed lengths of stay. In the Results section, we further report a smoothed AUROC over time using the nearest-neighbor estimator for time-dependent ROC curves [54]. Our code is available in the official code repository [55].

## Results

### Study Setting

**Figure 2:**
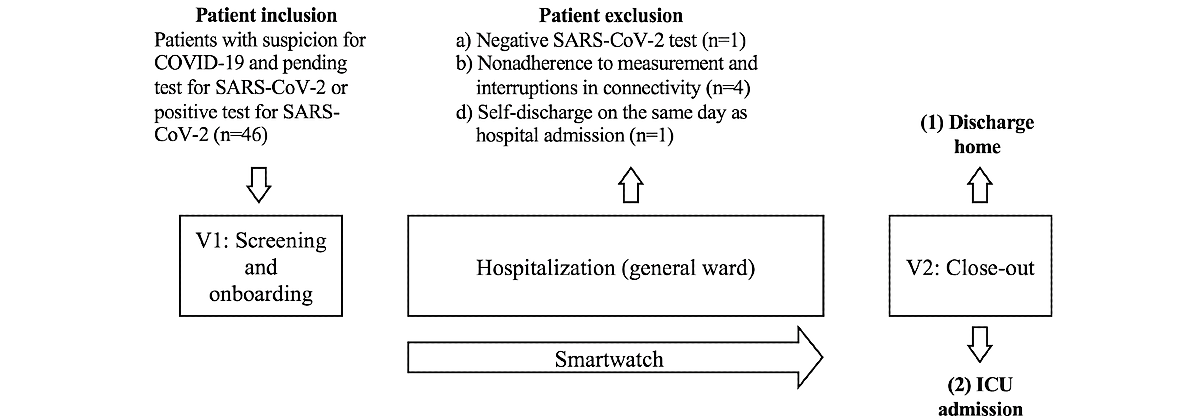
Overview of study with a study flowchart. Data were obtained according to the study flowchart. During visit 1 (V1), 46 eligible patients were recruited. After hospitalization in the general ward, patients were equipped with a consumer-grade wearable (smartwatch). We excluded patients with suspected COVID-19 in the case of a negative SARS-CoV-2 test (n=1). In addition, patients were excluded due to nonadherence to measurement principles or interruptions in connectivity (n=4) and self-discharge on the same day as hospital admission (n=1). During visit 2 (V2), we recorded the patient outcomes (ie, discharge, n=31, vs ICU admission, n=7). Patients with unknown outcomes were right-censored (n=2). ICU: intensive care unit.

We conducted an observational study (see the study flowchart in Figure 2) between October 2020 and June 2021 in the general ward of a tertiary referral center in Switzerland. In total, 46 patients were recruited according to 2 different scenarios of recruitment and enrollment in the study: (1) Patients who attended the emergency ward and were hospitalized with suspicion of COVID-19 were recruited directly during their initial evaluation, and (2) additionally, all inpatients tested positive for SARS-CoV-2 were reported to the study team automatically with an email alert from the laboratory and thereafter contacted (in-hospital visit) by a member of the study team. Either of the following patient outcomes were possible: (1) hospital discharge or (2) ICU admission.

Inclusion criteria were age greater than 18 years, suspicion of COVID-19 or patient testing positive for SARS-CoV-2, and hospitalization in the general ward. Exclusion criteria were direct transfer from an emergency ward or external institution to the ICU (ie, no hospitalization in the general ward of the study institution). Further exclusion criteria were that the smartwatch could not be attached around the wrist of the patient, known allergies to components of the smartwatch, and rejection of ICU admission in the patient decree.

After screening, 1 (2.2%) of 46 individuals was excluded due to a negative SARS-CoV-2 result, 4 (8.7%) patients were excluded due to technical problems during the recording (eg, persistent interruptions of the Bluetooth connection between wearable and smartphone) or nonadherence to the prescribed measurement regime, and 1 (2.2%) individual was excluded because the hospital discharge occurred on the same day of hospitalization. In total, 40 (87%) patients remained. Of these, 7 (17.5%) were admitted to an ICU during their hospital stay (after a median of 2 days), and 31 (77.5%) were discharged without a subsequent ICU stay (after a median of 4 days). In addition, 2 (5%) patients dropped out before their outcome was recorded and were thus treated as right-censored in our analysis.

### Association of Physiological Features With Patient Outcomes

**Figure 3:**
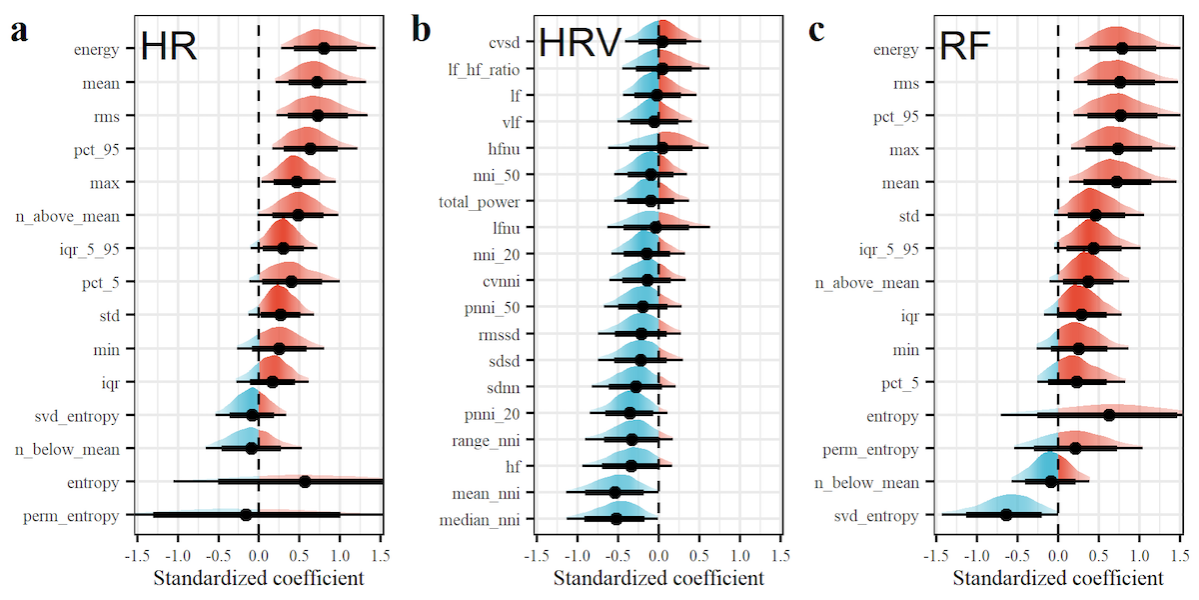
Association of physiological features with patient outcomes. Shown are the standardized coefficients of physiological features for the (a) HR, (b) HRV, and (c) RF. Features were computed based on daily physiological measurements from wearables (see the Feature Engineering section and Multimedia Appendix 1). For each coefficient, we reported the posterior probability mass with mean (dot) and the 80% and 95% CrIs (thick and thin bars, respectively). Positive values (red) indicate an association with a deterioration in the health condition, and negative values (blue) indicate an association with an improved health condition. CrI: credible interval; HR: heart rate; HRV: heart rate variability; RF: respiration frequency.

Figure 3 shows the association of physiological features with patient outcomes. Specifically, we reported standardized coefficients of the physiological features obtained from survival models adjusting for patient age and sex. A positive coefficient indicates that an increase in the value of a physiological feature on day “t” is associated with a higher probability of ICU admission as well as a lower probability of hospital discharge on day “t.” In contrast, a negative coefficient indicates that an increase in the value of a physiological feature is associated with a lower probability of ICU admission and a higher probability of hospital discharge on a given day.

Overall, 49 different univariate associations were estimated (ie, with 15, 30.6%, features related to the HR, 19, 38.8%, features related to the HRV, and 15, 30.6%, features related to the RF). For features related to the HR, we found the following associations: a higher HR was associated with worsened patient outcomes. In particular, we found that an increase in the mean HR indicated a deterioration in the patient’s condition (coefficient 0.71, 95% CrI 0.20-1.32). A similar association was found for several other features, including the maximum HR (coefficient 0.46, 95% CrI 0.03-0.94). For entropy-based features, the estimated relationship remained largely uncertain, however. For features related to the HRV, we found that increases were associated with improved patient outcomes. For example, an increase in the standard deviation of normal-to-normal intervals (SDNN) indicated an improvement of the patient condition (coefficient –0.28, 95% CrI –0.82 to 0.21). Moreover, several features related to the RF showed a positive association, where larger values indicated a worsened patient outcome. For example, increases in the 95% quantile of the RF were associated with a deterioration in the patient’s condition (coefficient 0.77, 95% CrI 0.19-1.51). The same was observed for the RF SD (0.46, 95% CrI –0.05 to 1.06). Altogether, these associations establish that the risk of a worsened condition among inpatients with COVID-19 can be identified through health measurements from consumer-grade wearables.

As part of our robustness checks, various alternative model specifications were tested (ie, changes with respect to the time window used for physiological measurements, the time trend, subject-specific variation, the cumulative distribution function, and wider priors; see Multimedia Appendix 4). We obtained similar estimates for all models, thus implying that the estimated associations between physiological features and patient outcomes remain robust.

### Development of a Risk Score

Next, the physiological measurements from the wearables were combined into an overall risk score. Here, our main aim was to demonstrate that a combination of different physiological features is of predictive value and thus jointly informative. For this, PCA [45] was applied to all features that showed a relevant association (80% CrI, excluding 0) with patient outcomes. This was the case for 9 HR features, 4 HRV features, and 9 RF features. Next, PCs with the highest predictive value for patient outcomes were identified using LASSO (see the Methods section). For our clinical data, the LASSO selected 8 (36.4%) of 22 PCs. These PCs characterized the physiological state of patients through a lower-dimensional representation of the wearable-based measurements. A visualization of the PCs is shown in Multimedia Appendix 5.

**Figure 4:**
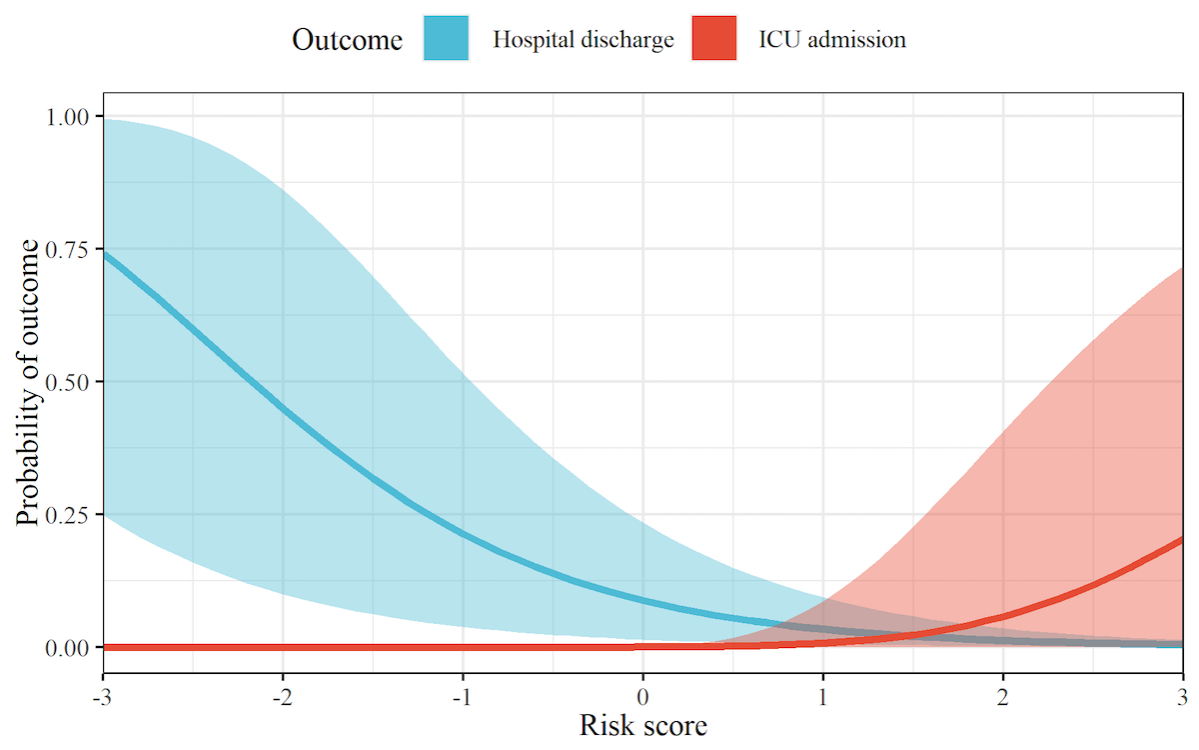
Probability of hospital discharge and ICU admission for different values of the risk score. Shown is the estimated daily probability of hospital discharge (blue) and ICU admission (red) as a function of the risk score. A larger risk score implies a higher probability of ICU admission and a lower probability of hospital discharge. Posterior means (lines) and 95% CrIs (shaded areas) are reported. The probability of continued stay (ie, neither hospital discharge nor ICU admission) is not shown but can be computed as P_continued stay_ = 1 – P_discharge_ – P_ICU_. CrI: credible interval; ICU: intensive care unit.

The selected PCs were used to model patient outcomes as the dependent variable based on a survival model that is similar to that of the explanatory analysis. Multimedia Appendix 5 reports the estimated coefficients. The resulting linear predictor for the probability of hospital discharge and ICU admission was used as the overall risk score. The risk score thus quantifies the probability of hospital discharge and ICU admission of patients on a given day using wearable-based measurements from the previous night. Here, a higher score generally indicates a worse patient condition (Figure 4). Although the overall health condition of patients is confidently predicted by the risk score, the smaller number of patients with ICU admission in our data set means that the risk score is less differentiated with regard to ICU admission.

### Evaluation of the Risk Score

**Figure 5:**
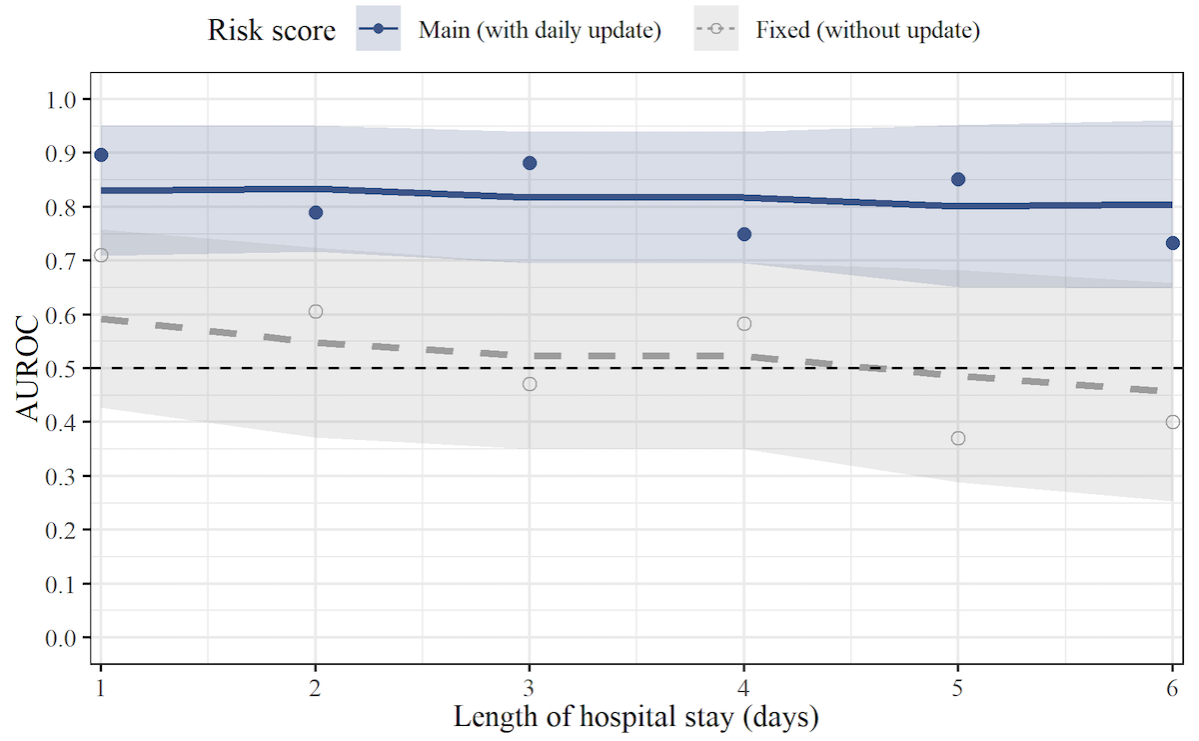
Prediction performance of the risk score over time. Shown is the time-dependent AUROC of the risk score in predicting patient discharge over time. Two scenarios are compared: (1) main (blue solid line) and (2) fixed (gray dashed line). In the main scenario, the daily risk score is computed from updated wearable-based measurements recorded during the respective previous night. The AUROC is significantly above 0.5 for up to 6 days, which covers 87% of the patients’ length of stay. In the fixed scenario, the risk score is computed throughout the stay from recordings only from the first night. The comparison between these scenarios shows the added value of regularly updated health measurements provided by wearables. Out-of-sample predictions were obtained via leave-one-patient-out cross-validation. Dots show the individual time-dependent AUROC estimates for days with observed patient discharge. Smoothing was performed via a nearest-neighbor estimator (see the Performance Evaluation section) to obtain an estimate of the mean AUROC over time (lines) with 95% CIs (shaded areas). AUROC: area under the receiver operating characteristic curve.

Figure 5 shows the leave-one-patient-out cross-validation results for the predictive performance of our risk score. Because the risk score was updated as the condition of patients changed throughout their hospital stay, the time-dependent AUROC was used as a performance metric that accounts for time-varying prediction performance. A daily AUROC was computed for up to 6 days of hospital stay, which covered 87% of the patients’ length of stay. For different lengths of stay, the risk score achieved a time-dependent AUROC of 0.73-0.90, suggesting reasonable predictive performance (Figure 5). This establishes that the different physiological features are jointly informative of patient health condition over time.

For comparison, we also reported the performance of a fixed risk score that used only data from the first night of hospital stay but was otherwise identical (Figure 5). Comparing the performance of the fixed risk score and our original risk score allowed us to assess the benefit of daily updating the physiological measurements. For the first day of hospital stay, the fixed risk score achieved a performance that was worse than the risk score with updated physiological measurements but was still above 0.70. However, for a length of stay longer than 1 day, the fixed risk score showed a consistently inferior performance.

To further assess the added value of the physiological features used in our risk score, we also evaluated the performance of a risk score that uses demographic features (ie, patient age and sex) but no physiological features. The cross-validation results for this risk score indicated that demographic features alone have no relevant predictive value with regard to the time-varying health condition of the patients in our sample (see Multimedia Appendix 6). Together, these results confirm that continuously updated, repeated monitoring of physiological measurements can provide an added value for analyzing the patient’s condition during the hospital stay.

## Discussion

### Principal Results

This work presents a monitoring system that allows for risk scoring of inpatients with COVID-19 in the general ward using consumer-grade wearables (smartwatches).

For this, Bayesian survival analysis was used to establish that physiological measurements monitored by consumer-grade wearables are indicative of patient outcomes in the general ward (ie, hospital discharge vs ICU admission). We further showed that these different physiological measurements can be combined into a single, clinically meaningful risk score with high prediction performance regarding the health condition of patients (time-dependent AUROC of 0.73-0.90). Our results show the feasibility of a risk score for inpatients with COVID-19 in general wards based on scalable consumer-grade wearables. In the future, such risk scores may enable clinical practitioners to adapt to patient needs and, ideally, respond earlier when a patient trajectory progresses toward a critical condition.

We found that several physiological features derived from wearable-based measurements are associated with patient outcomes. For instance, a higher mean HR, a higher mean RF, and a lower HRV RMSSD are all indicative of a deterioration in the health condition of patients. The observed relationship between patient outcomes and cardiovascular features (HR and HRV), as well as patient outcomes and RF measurements, is consistent with previous research on digital biomarkers [22,56-60]. These findings add to the robustness of our monitoring system. Importantly, we discovered these associations based on consumer-grade wearables, which indicates the clinical applicability and, thus, the relevance of the technology. Furthermore, the risk score may implicitly capture information on clinical interventions (eg, ventilation, which affects the RF). Hence, wearable recordings must be interpreted carefully in the light of other simultaneous interventions.

To derive our risk score, we intentionally chose a parsimonious approach using feature engineering and Bayesian survival modeling. Different from other machine learning methods, a parametric, Bayesian approach like ours is especially viable in the case of newly emerged diseases, where data availability may be limited. Since our feature engineering is mostly disease independent, the physiological features could be integrated into models for other diseases too, further promoting scalability. More generally, our approach demonstrates how multiple competing patient outcomes can be flexibly linked to time-dependent measurements in a parametric, joint model of patient condition.

### Comparison With Prior Work

Prior research successfully explored vital signs measured by smartwatches (eg, the resting HR) as a basis to detect the onset of COVID-19 outside a clinical setting [12-14]. Hence, we leveraged similar devices to record physiological measures and model a similar outcome (ie, deterioration in a patient's health). However, our monitoring system differs from others on COVID-19 as follows: First, there is no proof-of-concept study in a clinical setup that explores smartwatches as a basis to monitor patients with COVID-19 to the best of our knowledge. Second, we modeled a patient's health condition as a whole to detect not only a deterioration in the patient's health but also an improvement.

Lastly, several studies have focused on risk scoring in ICUs [7,8,16,17]. However, due to a large number of hospitalizations for COVID-19, inpatients in general wards are also of major concern. Different from our study setting, risk scoring in ICUs builds upon specialized medical devices for health monitoring and a specific patient population. Because of this, a direct transfer of ICU risk scores to clinical practice in general wards is obviously limited. Therefore, we developed a monitoring system and subsequent risk scoring that is particularly suited for general wards (eg, there is no need for specialized medical monitoring technology).

In summary, our study supports the clinical relevance of wearables exclusively based on consumer-grade technology. In contrast to specialized medical devices for health monitoring (eg, finger pulse oximetry or ECG sensor), consumer-grade technology comes at a comparatively low cost, can be deployed easily, and is thus scalable. Clinical practitioners simply need to attach the smartwatch to the wrist of a patient. In addition, smartwatches offer a familiar user interface.

### Limitations

A general concern may be that measurements from consumer-grade wearables are subject to noise or missing values. The results of our study, however, show that a wearable-based risk score can offer robust predictions of patient outcomes. Our study opens several possibilities for future research. The main limiting factor of our study is the sample size of 40, which naturally restricts the number of ICU admissions in the data set. To further assess the predictive performance of wearable-based risk scores, in particular with regard to ICU admission, future research might expand our data set with additional patient populations and different variants of SARS-CoV-2. The model merely incorporated data from wearable sensors for risk scoring and refrained from integrating other data sources (eg, electronic health records). This choice was made to ensure a scalable use in clinical practice. Further, our system builds upon dimensionality reduction via PCA to handle high-dimensional sensor data, proving effective to avoid overfitting. Nevertheless, future research may explore alternative machine learning methods for risk scoring.

### Conclusion

Overall, our results show the promise of consumer-grade wearables as an effective, scalable, and low-cost technology for health monitoring in a general ward. In the future, consumer-grade wearables, such as smartwatches, may further offer monitoring capabilities for inpatients with other diseases.

## Appendix

Multimedia Appendix 1 Data description.

Multimedia Appendix 2 Method details.

Multimedia Appendix 3 Model Diagnostics.

Multimedia Appendix 4 Robustness of Explanatory Analysis.

Multimedia Appendix 5 Principal component analysis (PCA) results.

Multimedia Appendix 6 Comparison to a Risk Score Using Only Demographic Features.

## Abbreviations

ACC: accelerometer
AUROC: area under the receiver operating characteristic curve
CrI: credible interval
ECG: electrocardiogram
HRV: heart rate variability
HR: heart rate
HRV: heart rate variability
IBI: interbeat interval
ICU: intensive care unit
LASSO: least absolute shrinkage and selection operator
PC: principal component
PCA: principal component analysis
PPG: photoplethysmography
RF: respiration frequency
ROC: receiver operating characteristic

## References

[1] Bolourani S, Brenner M, Wang P, McGinn T, Hirsch JS, Barnaby D, Zanos TP, Northwell COVID-19 Research Consortium (2021). A machine learning prediction model of respiratory failure within 48 hours of patient admission for COVID-19: model development and validation. J Med Internet Res.

[2] García LF (2020). Immune response, inflammation, and the clinical spectrum of COVID-19. Front Immunol.

[3] Jose RJ, Manuel A (2020). COVID-19 cytokine storm: the interplay between inflammation and coagulation. Lancet Respir Med.

[4] Razavian N, Major VJ, Sudarshan M, Burk-Rafel J, Stella P, Randhawa H, Bilaloglu S, Chen J, Nguy V, Wang W, Zhang H, Reinstein I, Kudlowitz D, Zenger C, Cao M, Zhang R, Dogra S, Harish KB, Bosworth B, Francois F, Horwitz LI, Ranganath R, Austrian J, Aphinyanaphongs Y (2020). A validated, real-time prediction model for favorable outcomes in hospitalized COVID-19 patients. NPJ Digit Med.

[5] Cummings BC, Ansari S, Motyka JR, Wang G, Medlin RP, Kronick SL, Singh K, Park PK, Napolitano LM, Dickson RP, Mathis MR, Sjoding MW, Admon AJ, Blank R, McSparron JI, Ward KR, Gillies CE (2021). Predicting intensive care transfers and other unforeseen events: analytic model validation study and comparison to existing methods. JMIR Med Inform.

[6] Schwab P, Mehrjou A, Parbhoo S, Celi LA, Hetzel J, Hofer M, Schölkopf B, Bauer S (2021). Real-time prediction of COVID-19 related mortality using electronic health records. Nat Commun.

[7] Zhao Z, Chen A, Hou W, Graham JM, Li H, Richman PS, Thode HC, Singer AJ, Duong TQ (2020). Prediction model and risk scores of ICU admission and mortality in COVID-19. PLoS One.

[8] Subudhi S, Verma A, Patel AB, Hardin CC, Khandekar MJ, Lee H, McEvoy D, Stylianopoulos T, Munn LL, Dutta S, Jain RK (2021). Comparing machine learning algorithms for predicting ICU admission and mortality in COVID-19. NPJ Digit Med.

[9] Cho S, Park S, Song M, Bae YY, Lee D, Kim D (2021). Prognosis score system to predict survival for COVID-19 cases: a Korean nationwide cohort study. J Med Internet Res.

[10] Emanuel EJ, Persad G, Upshur R, Thome B, Parker M, Glickman A, Zhang C, Boyle C, Smith M, Phillips JP (2020). Fair allocation of scarce medical resources in the time of Covid-19. N Engl J Med.

[11] Truog RD, Mitchell C, Daley GQ (2020). The toughest triage - allocating ventilators in a pandemic. N Engl J Med.

[12] Quer G, Radin JM, Gadaleta M, Baca-Motes K, Ariniello L, Ramos E, Kheterpal V, Topol EJ, Steinhubl SR (2021). Wearable sensor data and self-reported symptoms for COVID-19 detection. Nat Med.

[13] Mishra T, Wang M, Metwally AA, Bogu GK, Brooks AW, Bahmani A, Alavi A, Celli A, Higgs E, Dagan-Rosenfeld O, Fay B, Kirkpatrick S, Kellogg R, Gibson M, Wang T, Hunting EM, Mamic P, Ganz AB, Rolnik B, Li X, Snyder MP (2020). Pre-symptomatic detection of COVID-19 from smartwatch data. Nat Biomed Eng.

[14] Menni C, Valdes AM, Freidin MB, Sudre CH, Nguyen LH, Drew DA, Ganesh S, Varsavsky T, Cardoso MJ, El-Sayed Moustafa JS, Visconti A, Hysi P, Bowyer RCE, Mangino M, Falchi M, Wolf J, Ourselin S, Chan AT, Steves CJ, Spector TD (2020). Real-time tracking of self-reported symptoms to predict potential COVID-19. Nat Med.

[15] Pan P, Li Y, Xiao Y, Han B, Su L, Su M, Li Y, Zhang S, Jiang D, Chen X, Zhou F, Ma L, Bao P, Xie L (2020). Prognostic assessment of COVID-19 in the intensive care unit by machine learning methods: model development and validation. J Med Internet Res.

[16] Cheng F, Joshi H, Tandon P, Freeman R, Reich DL, Mazumdar M, Kohli-Seth R, Levin M, Timsina P, Kia A (2020). Using machine learning to predict ICU transfer in hospitalized COVID-19 patients. J Clin Med.

[17] Galloway JB, Norton S, Barker RD, Brookes A, Carey I, Clarke BD, Jina R, Reid C, Russell MD, Sneep R, Sugarman L, Williams S, Yates M, Teo J, Shah AM, Cantle F (2020). A clinical risk score to identify patients with COVID-19 at high risk of critical care admission or death: an observational cohort study. J Infect.

[18] Nelson BW, Allen NB (2019). Accuracy of consumer wearable heart rate measurement during an ecologically valid 24-hour period: intraindividual validation study. JMIR Mhealth Uhealth.

[19] Dunn J, Kidzinski L, Runge R, Witt D, Hicks JL, Schüssler-Fiorenza Rose SM, Li X, Bahmani A, Delp SL, Hastie T, Snyder MP (2021). Wearable sensors enable personalized predictions of clinical laboratory measurements. Nat Med.

[20] Bent B, Cho PJ, Henriquez M, Wittmann A, Thacker C, Feinglos M, Crowley MJ, Dunn JP (2021). Engineering digital biomarkers of interstitial glucose from noninvasive smartwatches. NPJ Digit Med.

[21] Maritsch M, Föll S, Lehmann V, Bérubé C, Kraus M, Feuerriegel S, Kowatsch T, Züger T, Stettler C, Fleisch E, Wortmann F (2020). Towards wearable-based hypoglycemia detection and warning in diabetes. https://linkinghub.elsevier.com/retrieve/pii/S0169-2607(21)00535-6.

[22] Natarajan A, Su H, Heneghan C (2020). Assessment of physiological signs associated with COVID-19 measured using wearable devices. NPJ Digit Med.

[23] Orphanidou C (2018). Signal Quality Assessment in Physiological Monitoring. State of the Art and Practical Considerations.

[24] Lu S, Zhao H, Ju K, Shin K, Lee M, Shelley K, Chon KH (2008). Can photoplethysmography variability serve as an alternative approach to obtain heart rate variability information?. J Clin Monit Comput.

[25] Malik M, Bigger JT, Camm AJ, Kleiger RE, Malliani A, Moss AJ, Schwartz PJ (1996). Heart rate variability: standards of measurement, physiological interpretation, and clinical use. Eur Heart J.

[26] Bent B, Goldstein BA, Kibbe WA, Dunn JP (2020). Investigating sources of inaccuracy in wearable optical heart rate sensors. NPJ Digit Med.

[27] Bachler M (2017). Spectral analysis of unevenly spaced data: models and application in heart rate variability. SNE.

[28] Schaffer T, Hensel B, Weigand C, Schüttler J, Jeleazcov C (2014). Evaluation of techniques for estimating the power spectral density of RR-intervals under paced respiration conditions. J Clin Monit Comput.

[29] Laguna P, Moody G, Mark R (1998). Power spectral density of unevenly sampled data by least-square analysis: performance and application to heart rate signals. IEEE Trans Biomed Eng.

[30] Morelli D, Rossi A, Cairo M, Clifton DA (2019). Analysis of the impact of interpolation methods of missing RR-intervals caused by motion artifacts on HRV features estimations. Sensors (Basel).

[31] Lomb NR (1976). Least-squares frequency analysis of unequally spaced data. Astrophys Space Sci.

[32] Moody G (1993). Spectral analysis of heart rate without resampling.

[33] Acharya RU, Joseph PK, Kannathal N, Lim CM, Suri JS (2006). Heart rate variability: a review. Med Biol Eng Comput.

[34] Shaffer F, Ginsberg JP (2017). An overview of heart rate variability metrics and norms. Front Public Health.

[35] Spiers JP, Silke B, McDermott U, Shanks RG, Harron DWG (1993). Time and frequency domain assessment of heart rate variability: a theoretical and clinical appreciation. Clin Auton Res.

[36] Mietus JE, Peng C-k, Henry I, Goldsmith RL, Goldberger AL (2002). The pNNx files: re-examining a widely used heart rate variability measure. Heart.

[37] Föll S, Maritsch M, Spinola F, Mishra V, Barata F, Kowatsch T, Fleisch E, Wortmann F (2021). FLIRT: a feature generation toolkit for wearable data. Comput Methods Programs Biomed.

[38] D'Agostino RB, Lee M, Belanger AJ, Cupples LA, Anderson K, Kannel WB (1990). Relation of pooled logistic regression to time dependent Cox regression analysis: the Framingham Heart Study. Stat Med.

[39] Ngwa JS, Cabral HJ, Cheng DM, Pencina MJ, Gagnon DR, LaValley MP, Cupples LA (2016). A comparison of time dependent Cox regression, pooled logistic regression and cross sectional pooling with simulations and an application to the Framingham Heart Study. BMC Med Res Methodol.

[40] Austin PC, Lee DS, Fine JP (2016). Introduction to the analysis of survival data in the presence of competing risks. Circulation.

[41] Agresti A (2010). Analysis of Ordinal Categorical Data, Second Edition.

[42] McCullagh P (2018). Regression models for ordinal data. J R Stats Soc (Methodol).

[43] Bürkner P, Vuorre M (2019). Ordinal regression models in psychology: a tutorial. Adv Methods Pract Psychol Sci.

[44] Cox DR (2018). Regression models and life-tables. J R Stats Soc (Methodol).

[45] Hotelling H (1933). Analysis of a complex of statistical variables into principal components. J Educ Psychol.

[46] Gelman A, Rubin D, Carlin J, Stern H (2021). Bayesian Data Analysis. 1st ed.

[47] Bürkner P (2017). brms: an R package for Bayesian multilevel models using Stan. J Stats Softw.

[48] Hoffman M, Gelman A (2014). The No-U-Turn sampler: adaptively setting path lengths in Hamiltonian Monte Carlo. J Mach Learn Res.

[49] Stan Development Team Stan Language Reference Manual (Version 2.25).

[50] Stan Development Team Prior Choice Recommendations.

[51] Heagerty PJ, Zheng Y (2005). Survival model predictive accuracy and ROC curves. Biometrics.

[52] Saha P, Heagerty PJ (2010). Time-dependent predictive accuracy in the presence of competing risks. Biometrics.

[53] Bansal A, Heagerty PJ (2018). A tutorial on evaluating the time-varying discrimination accuracy of survival models used in dynamic decision making. Med Decis Making.

[54] Heagerty P J, Lumley T, Pepe M S (2000). Time-dependent ROC curves for censored survival data and a diagnostic marker. Biometrics.

[55] (2021). Official code repository.

[56] Bhatraju PK, Ghassemieh BJ, Nichols M, Kim R, Jerome KR, Nalla AK, Greninger AL, Pipavath S, Wurfel MM, Evans L, Kritek PA, West TE, Luks A, Gerbino A, Dale CR, Goldman JD, O'Mahony S, Mikacenic C (2020). Covid-19 in critically ill patients in the Seattle region - case series. N Engl J Med.

[57] Hirten RP, Danieletto M, Tomalin L, Choi KH, Zweig M, Golden E, Kaur S, Helmus D, Biello A, Pyzik R, Charney A, Miotto R, Glicksberg BS, Levin M, Nabeel I, Aberg J, Reich D, Charney D, Bottinger EP, Keefer L, Suarez-Farinas M, Nadkarni GN, Fayad ZA (2021). Use of physiological data from a wearable device to identify SARS-CoV-2 infection and symptoms and predict COVID-19 diagnosis: observational study. J Med Internet Res.

[58] Hasty F, García G, Dávila CH, Wittels S, Hendricks S, Chong S (2020). Heart rate variability as a possible predictive marker for acute inflammatory response in COVID-19 patients. Mil Med.

[59] Massaroni C, Nicolò A, Schena E, Sacchetti M (2020). Remote respiratory monitoring in the time of COVID-19. Front Physiol.

[60] Xu J, Wang W, Ye H, Pang W, Pang P, Tang M, Xie F, Li Z, Li B, Liang A, Zhuang J, Yang J, Zhang C, Ren J, Tian L, Li Z, Xia J, Gale RP, Shan H, Liang Y (2021). A predictive score for progression of COVID-19 in hospitalized persons: a cohort study. NPJ Prim Care Respir Med.

