# Supplementary material for: A Scalable Risk-Scoring System Based on Consumer-Grade Wearables for Inpatients With COVID-19: Statistical Analysis and Model Development
Source: JMIR Form Res. 2022 Jun 21;6(6):e35717. doi: 10.2196/35717 (PMC9217156; doi:10.2196/35717)
Supplement: Multimedia Appendix 1 [file formative_v6i6e35717_app1.docx]

# Multimedia Appendix 1. Data Description

## A Data

As a preparation for our clinical study, we anticipated an enrollment of 100 patients based on the methodology provided by Obuchowski, Lieber, and Wians (2004) [1]. The study was terminated at the end of the 3rd COVID-19 wave in Switzerland when no new patients could be recruited. Nevertheless, we were able to recruit 46 patients. We excluded patients with suspected COVID-19 in case of a negative SARS-CoV-2 test (*n* = 1). In addition, patients were excluded due to non-adherence to measurement principles or interruptions in connectivity (*n* = 4), and self-discharge on the same day as hospital admission (*n* = 1). Overall, we used data from 40 patients to validate our risk score.

### A.1 Demographics

Table 1. Patient characteristics ($N=40$)

| **Outcome type** | **Variable** | **Distribution (mean ± SD)** |
| --- | --- | --- |
|  |  |  |
| **Hospital discharge (**$\boldsymbol{n=31}$**)** |  |  |
|  | Age [years] | 55.81 ± 15.02 |
|  | Sex | 10 female, 21 male |
| **ICU admission (**$\boldsymbol{n=7)}$ |  |  |
|  | Age [years] | 59.29 ± 16.12 |
|  | Sex | 1 female, 6 male |
| **Dropout (**$\boldsymbol{n=2}$**)** |  |  |
|  | Age [years] | 70.00 ± 4.24 |
|  | Sex | 1 female, 1 male |

### A.2 Physiological Features

Table 2. Overview of aggregations used in feature engineering for physiological features.^a^

| **Measurement** | **Aggregation** | **Description** | **Unit** |
| --- | --- | --- | --- |
|  |  |  |  |
| **HR, RF** |  |  |  |
|  | mean | Mean of the signal | BePM, BrPM |
|  | STD | Standard deviation of the signal | BePM, BrPM |
|  | min/max | Minimum and maximum of the signal | BePM, BrPM |
|  | energy | Energy of the signal | BePM^2^, BrPM^2^ |
|  | RMS | Root mean square of the signal | BePM, BrPM |
|  | n_above mean_ | Number of signal above the mean | – |
|  | n_below mean_ | Number of signal below the mean | – |
|  | IQR | Interquartile range between the 25th and 75th percentile of the signal | BePM, BrPM |
|  | IQR_5-95_ | Interquantile range between the 5th and 95th percentile of the signal | BePM, BrPM |
|  | PCT_5_ | 5th percentile of the signal | – |
|  | PCT_95_ | 95th percentile of the signal | – |
|  | entropy | Entropy of the signal | – |
|  | entropy_PER_ | Permutation entropy of the signal | – |
|  | entropy_SVD_ | Singular value decomposition of the signal entropy | – |
| **HRV** |  |  |  |
|  | RMSSD | Root mean square of successive differences | ms |
|  | SDNN | Standard deviation of normal-to-normal interval | ms |
|  | SDSD | Standard deviation of successive differences | ms |
|  | PNN_20_ | Proportion of successive normal-to-normal interval differences exceeding 20 ms | % |
|  | NNI_20_ | Proportion of successive normal-to-normal differing by more than 20 ms in the entire recording | ms |
|  | PNN_50_ | Number of pairs of adjacent normal-to-normal interval differences exceeding 50 ms | % |
|  | NNI_50_ | Proportion of successive normal-to-normal differing by more than 50 ms in the entire recording | ms |
|  | CVNN | Coefficient of variation equal to the ratio of SDNN divided by mean normal-to-normal interval | - |
|  | CVSD | Coefficient of variation of successive differences equal to the RMSSD divided by mean normal-to-normal interval | - |
|  | median_NNI_ | Median of the normal-to-normal intervals | ms |
|  | mean_NNI_ | Mean of the normal-to-normal intervals | ms |
|  | range_NNI_ | Range of the normal-to-normal intervals | ms |
|  | total power | The variance of normal-to-normal intervals over the temporal segment below 0.04 Hz | ms^2^ |
|  | VLF | Power in very low frequency range below or equal 0.04  Hz | ms^2^ |
|  | LF | Power in low frequency range 0.04 Hz and 0.15 Hz | ms^2^ |
|  | HF | Power in high frequency range 0.15 Hz and 0.4 Hz | ms^2^ |
|  | LF/HF-ratio | Ratio of LF to HF | - |
|  | LFNU | LF power in normalized units | - |
|  | HFNU | HF power in normalized units | - |

^a^Described are the physiological features generated via feature engineering from from heart rate (HR) in beats per minute (BePM), heart rate variability (HRV) in milliseconds (ms), and respiration frequency (RF) in breaths per minute (BrPM) measurements.

### A.3 Intra-day Variability of Physiological Measurements

Physiological features also depend on the activity level and biological rhythm of patients and can thus vary throughout the day. Figure 6 shows standardized values of mean HR, HRV RMSSD, and mean RF throughout the day. Measurements of body movements from aggregated accelerometer data are also shown to indicate the activity level of patients. As can be seen, measurements are fairly stable during the phase of patients’ night rest, where body movements are comparatively low, but vary considerably during the day.

**
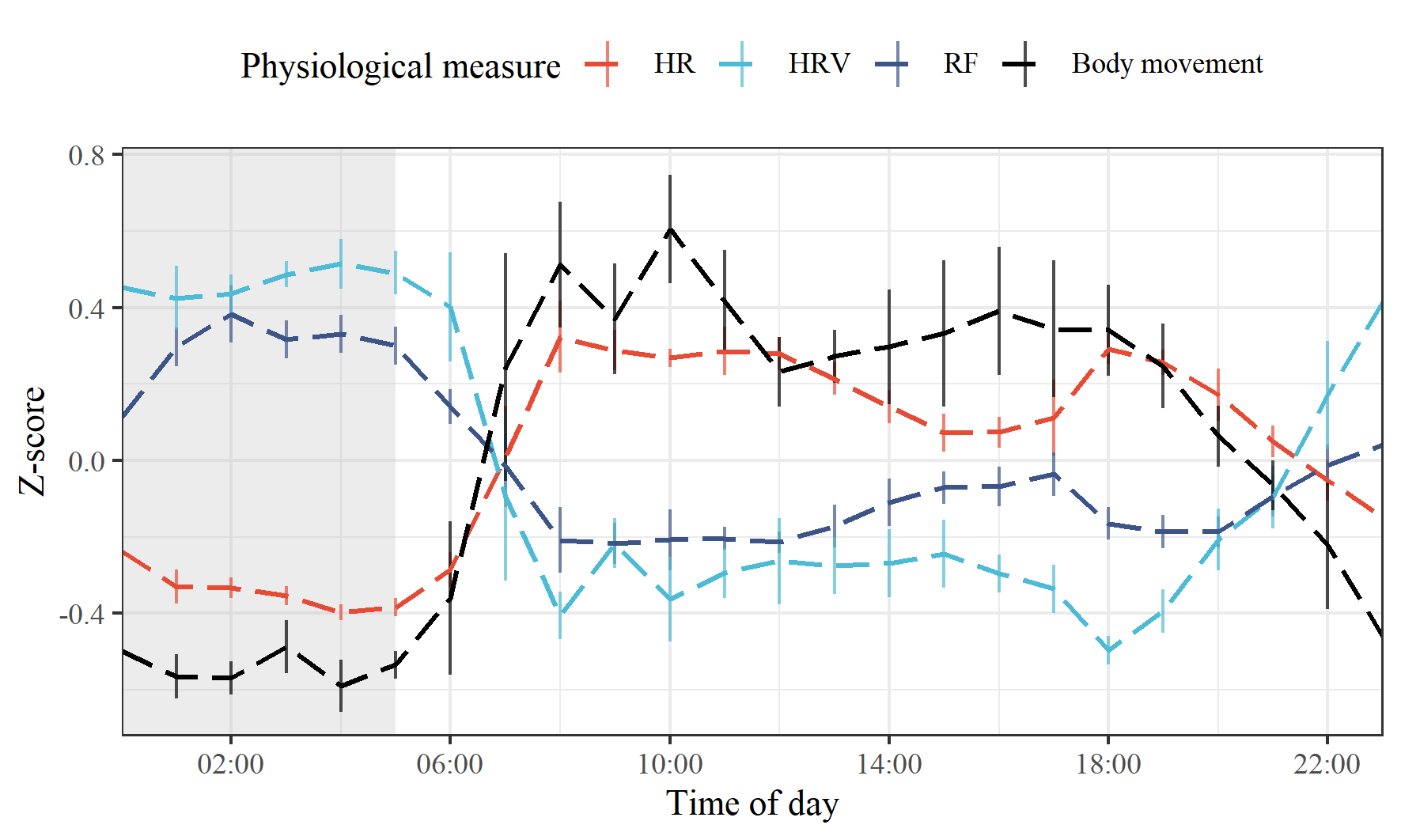
**

Figure 6. **Daily variation of physiological measurements and body movements.** Shown are the standardized values of 5-minute interval measurements of mean HR, HRV RMSSD, mean RF, and body movements, summarized for each hour of the day. Whiskers show the standard deviation for each hour. The time from 0:00a.m. – 5:00a.m. is highlighted in gray.

## References

1. Obuchowski NA, Lieber ML, Wians Jr. FH. ROC Curves in Clinical Chemistry: Uses, Misuses, and Possible Solutions. *Clinical Chemistry*. 2004;50(7):1118-1125. doi:10/bmzvpp
