# Supplementary material for: A Scalable Risk-Scoring System Based on Consumer-Grade Wearables for Inpatients With COVID-19: Statistical Analysis and Model Development
Source: JMIR Form Res. 2022 Jun 21;6(6):e35717. doi: 10.2196/35717 (PMC9217156; doi:10.2196/35717)
Supplement: Multimedia Appendix 2 [file formative_v6i6e35717_app2.docx]

# Multimedia Appendix 2

## B Method Details

### B.1 Model Specification

In the following, we present the formal specification of our survival models. We let $T_{i}$ denote the number of days until either hospital discharge or intensive care unit (ICU) admission of patient $i$ (i.e., time-to-first-event), where $\delta_{i}\in\{\text{discharge}, \text{icu}\}$ indicates the type of event. Then, the cause-specific hazards for patient $i$ on day $t$ were modeled as

|  | $\lambda_{\text{discharge}}\left( i,t \right)= P\left( T_{i}=t, \delta_{i}=\text{discharge} \mid t\leq T_{i},\eta_{i,t} \right)= F\left( \tau_{1}-\eta_{i,t} \right),$ | (1) |
| --- | --- | --- |
|  | $\lambda_{\text{icu}}\left( i,t \right)=P\left( T_{i}=t, \delta_{i}=\text{icu} \right\vert t\leq T_{i}, \eta_{i,t})=1-F(\tau_{2}-\eta_{i,t}),$ | (2) |

such that the probability of continued stay is given by

|  | $P\left( T_{i}>t \mid t\leq T_{i},\eta_{i,t} \right)=F\left( \tau_{2}-\eta_{i,t} \right)-F\left( \tau_{1}-\eta_{i,t} \right).$ | (3) |
| --- | --- | --- |

Here, $F(x)=1-e^{-e^{x}}$ is the cumulative distribution function of the extreme value distribution, $\tau_{1}$ and $\tau_{2}$ are ordinal threshold parameters, and $\eta_{i,t}$ is a linear predictor.

#### Explanatory Analysis

We let $x_{i,k,t}$ be the value of feature $k$ for patient $i$ on day $t$. Of note, the physiological features are time-dependent (indicated by subscript $t$). In our explanatory analysis, to estimate the association of physiological feature $k$ with patient health condition, we specified $\eta_{i,t}$ as

|  | $\eta_{i,t}^{\left( k \right)}=\alpha_{i}+\beta t+ \sum_{m} \delta^{(m)}z_{i,m}+ \theta^{(k)}x_{i,k,t} ,$ | (4) |
| --- | --- | --- |

where $\alpha_{i}$ is a random effect that captures variation between patients and where $\beta t$ is a linear trend that captures changes in the health condition over time. The term $\delta^{(m)}z_{i,m}$ adjusts for the baseline characteristic $m$ of patient $i$. We used patient age and sex as baseline characteristics. The parameter $\theta^{(k)}$ measures the conditional association of physiological feature $k$ with patient outcomes.

The above approach has a particular connection with the well-known proportional hazards model [44]. In the simpler case of single-type events with non-informative censoring and fixed covariates, pooled regression on daily event observations satisfies a discrete-time proportional hazards model [39, 1, 2]. Moreover, the use of a complementary log-log link gives rise to an extension of the continuous-time proportional hazards model, where survival times are grouped into discrete intervals [3]. Since time until hospital discharge or ICU admission has been indeed discretized into daily intervals in the present analysis, the choice of the extreme value distribution for our model is also theoretically justified. Overall, our approach can be interpreted as a Cox regression with time-varying covariates that further accounts for competing risks in a joint model of hospital discharge and ICU admission probability.

#### Risk Score

To compute our risk score, we used the linear predictor of a similar ordinal regression model as for the explanatory analysis, but with the selected principal components as covariates. Hence, the risk score $R(i,t)$ for patient $i$ on day $t$ is defined as

|  | $R(i,t)=\alpha_{i}+\beta t+ \sum_{m} \delta^{(m)}z_{i,m}+ \sum_{l} \theta^{(l)}q_{i,l,t} ,$ | (5) |
| --- | --- | --- |

where $q_{i,l,t}$ is the value of the $l$-th principal component of patient $i$ on day $t$.

To assess the added value of continuous physiological measurements for monitoring a patient’s health throughout their hospital stay, we compared our risk score model to an alternative model which uses only data from the first night of hospital stay but is otherwise identical. This “fixed” risk score was specified as

|  | $R^{\text{fixed}}(i,t)=\alpha_{i}+\beta t+ \sum_{m} \delta^{(m)}z_{i,m}+ \sum_{l} \theta^{(l)}q_{i,l}^{\text{fixed}} ,$ | (6) |
| --- | --- | --- |

where $q_{i,l}^{\text{fixed}}$ is the value of the $l$-th principal component for patient $i$ of physiological features obtained for the first night of hospital stay.

### B.2 Priors

In our models, we used weakly informative priors for all parameters (see Table 3), following general recommendations from the Stan development team [49].

Table 3. Prior choices for model parameters.^a^

| **Parameter** | **Description** | **Prior** | **Models** |
| --- | --- | --- | --- |
|  |  |  |  |
| $\alpha$ | Patient random effect | Normal($\mu= 0,\sigma= \sigma_{\alpha}$) | (I), (II) |
| $\sigma_{\alpha}$ | Standard deviation of $\alpha$ | Student-t^+^($\nu=3,\mu=0,\sigma=1$) | (I), (II) |
| $\beta$ | Time trend | Normal($\mu=0,\sigma=1$) | (I), (II) |
| $\delta^{(m)}$ | Demographic feature fixed effect | Normal($\mu=0,\sigma=1$) | (I), (II) |
| $\theta^{(k)}$ | Physiological feature fixed effect | Normal($\mu=0,\sigma=1$) | (I) |
| $\theta^{(l)}$ | Principal component fixed effect | Normal($\mu=0,\sigma=1$) | (II) |
| $\tau_{1},\tau_{2}$ | Ordinal threshold parameter | Normal($\mu=0,\sigma=5$) | (I), (II) |

^a^(I) refers to the explanatory models, (II) refers to the risk score models.

### B.3 Estimation

All model parameters were estimated using a fully Bayesian framework [46]. The estimation was conducted in R v4.0.3 using the package brms v2.15.0 and Markov chain Monte Carlo (MCMC) sampling via the No-U-Turn sampler (NUTS) [47, 48]. Four chains with 1000 warm-up iterations and 1000 sampling iterations each were run. All numeric variables were standardized prior to fitting, such that model parameters represent standardized coefficients. Unless stated otherwise, we report the posterior mean and the 95% CrI of estimated parameters. Weakly informative priors were used for all parameters, following general recommendations from the Stan development team (see Multimedia Appendix 2)[49].

The estimation was checked by following best-practice recommendations in Bayesian modeling [46, 50]. Specifically, the effective sample size and Gelman-Rubin convergence diagnostic were assessed for all parameters and the posterior predictive distribution of the model was inspected (see Multimedia Appendix 4). For all models, the diagnostics indicated a sufficient number of independent draws from the posterior distribution, mixing and convergence of the chains, and good model fit.

### B.3 Performance Evaluation

We evaluated the risk score-based prediction of the hazard of discharge $\hat{\lambda}_{\text{discharge}}\left( i,t \right)$ through the true positive rate (TP) for incident cases and the

false positive rate (FP) for dynamic controls, defined as

|  | $\text{T}\text{P}_{t}\left( c \right)=P(\hat{\lambda}_{\text{discharge}}\left( i,t \right)>c\mid T_{i}=t, Y_{i}=\text{discharge}) ,$ | (7) |
| --- | --- | --- |
|  | $\text{F}\text{P}_{t}\left( c \right)=P\left( \hat{\lambda}_{\text{discharge}}\left( i,t \right)>c \mid T_{i}>t \right) .$ | (8) |

Then, the time-dependent ROC curve and area under the ROC curve are

|  | $\text{ROC}_{t}\left( p \right)= \text{TP}_{t}\left\{ \left[ \text{FP}_{t} \right]^{-1}(p) \right\} ,$ | | (9) |
| --- | --- | --- | --- |
|  | $\text{AUROC}\left( t \right)= \int\text{RO}\text{C}_{t}\left( p \right) \text{d}p,$ | (10) | |

The time-dependent area under the receiver operating characteristic curve (AUROC) can be interpreted as the probability that a random patient $i$ who is discharged on day $t$ has a higher predicted hazard of discharge than a random patient $j$ who continues to stay in hospital [53]. Formally, this is given by

|  | $\text{AUROC}\left( t \right)= P\left( \hat{\lambda}_{\text{discharge}}\left( i,t \right)>\hat{\lambda}_{\text{discharge}}\left( j,t \right) \mid T_{i}=t, T_{j}>t \right) .$ | (11) |
| --- | --- | --- |

## References

1. Green MS, Symons MJ. A comparison of the logistic risk function and the proportional hazards model in prospective epidemiologic studies. *Journal of Chronic Diseases*. 1983;36(10):715-723. doi:10.1016/0021-9681(83)90165-0
2. Cupples LA, D’Agostino RB, Anderson K, Kannel WB. Comparison of baseline and repeated measure covariate techniques in the Framingham heart study. *Statistics in Medicine*. 1988;7(1-2):205-218. doi:10.1002/sim.4780070122
3. Kalbfleisch JD, Prentice RL. *The Statistical Analysis of Failure Time Data*. 2nd ed. John Wiley & Sons; 2002. doi:10.1002/9781118032985
