# Supplementary material for: A Scalable Risk-Scoring System Based on Consumer-Grade Wearables for Inpatients With COVID-19: Statistical Analysis and Model Development
Source: JMIR Form Res. 2022 Jun 21;6(6):e35717. doi: 10.2196/35717 (PMC9217156; doi:10.2196/35717)
Supplement: Multimedia Appendix 3 [file formative_v6i6e35717_app3.docx]

# Multimedia Appendix 3. Model Diagnostics.

## C Model Diagnostics

We followed best-practice recommendations in Bayesian modeling [46,50] and checked our estimation using appropriate model diagnostics. Specifically, we inspected the posterior predictive distribution of the model, the effective sample size, and the Gelman-Rubin convergence diagnostic for all parameters. The corresponding results are reported in the following. All checks had positive outcomes.

## C.1 Posterior Predictive Ability

We assessed the model fit by comparing the posterior predictive distribution of our models with the observed patient outcomes. For each model, we simulated 1000 draws from the posterior distribution of the model parameters to obtain predictions of hospital discharge and ICU admission for the patients in our dataset and compared the results with the true observations (Figure 7). The posterior predictive distributions matched the observations well, therefore implying good fit of all models. For reasons of brevity, the plots show only the fit of the models for mean HR, RMSSD (HRV), and mean RF. The posterior predictions from all other models were similar. In sum, this suggests a good fit of the models.


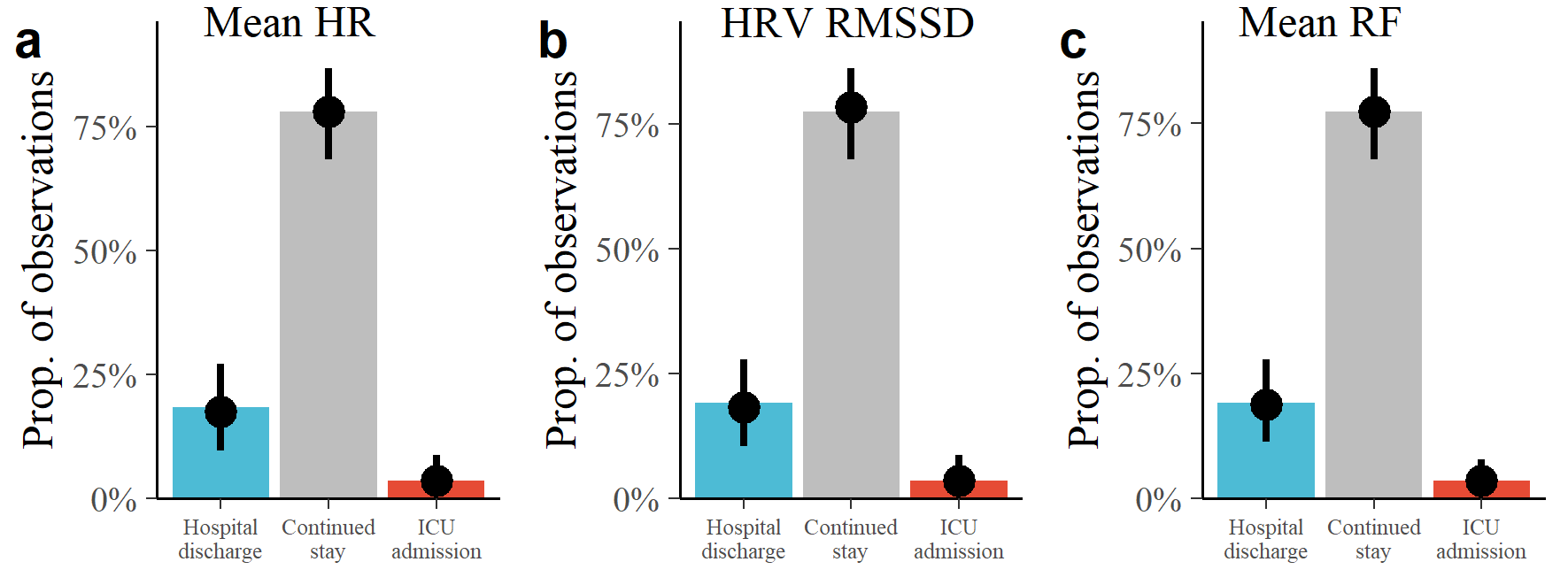
Figure 8: **Comparison of posterior predictive distribution with observed events.** Shown are exemplary posterior predictive checks for (a) mean HR, (b) RMSSD (HRV), and (c) mean RF. Shown are the means and 95% credible intervals of the posterior predictions (black) and the true proportions (colored) of hospital discharge, continued stay (no event), and ICU admission. The predictions agree well with the true proportions.

### C.2 Effective sample size

For each model parameter, we computed the ratio of the effective sample size to the total sample size [50] (Figure 8). The ratio was above 0.1 for all parameters, indicating a sufficient number of independent draws from the posterior distribution.


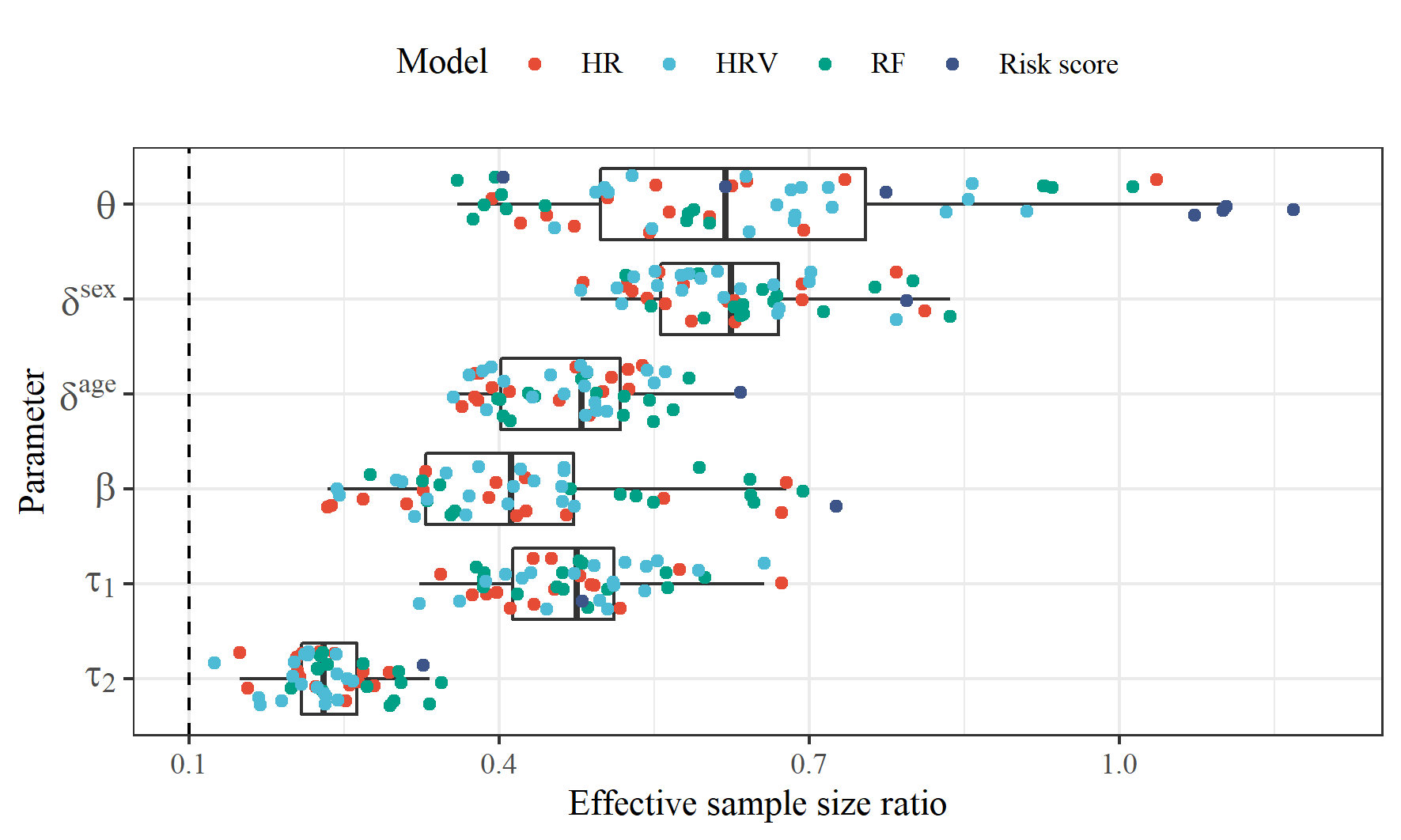


Figure 9: **Ratio of the effective sample size to the total sample size.** Shown are the ratios of the effective sample size to the total sample size (dots and boxplots) for parameters of the risk score model and the explanatory models. There were no values below 0.1 (i.e., fewer than 400 effective samples, which is generally considered problematic), implying a sufficient sample size.

## C.3 Convergence of Markov chains

To assess convergence of the Markov chains, we computed the Gelman-Rubin convergence diagnostic $\hat{R}$ [1] (Figure 9). The $\hat{R}$ values for all model parameters were below 1.01, indicating convergence [46].


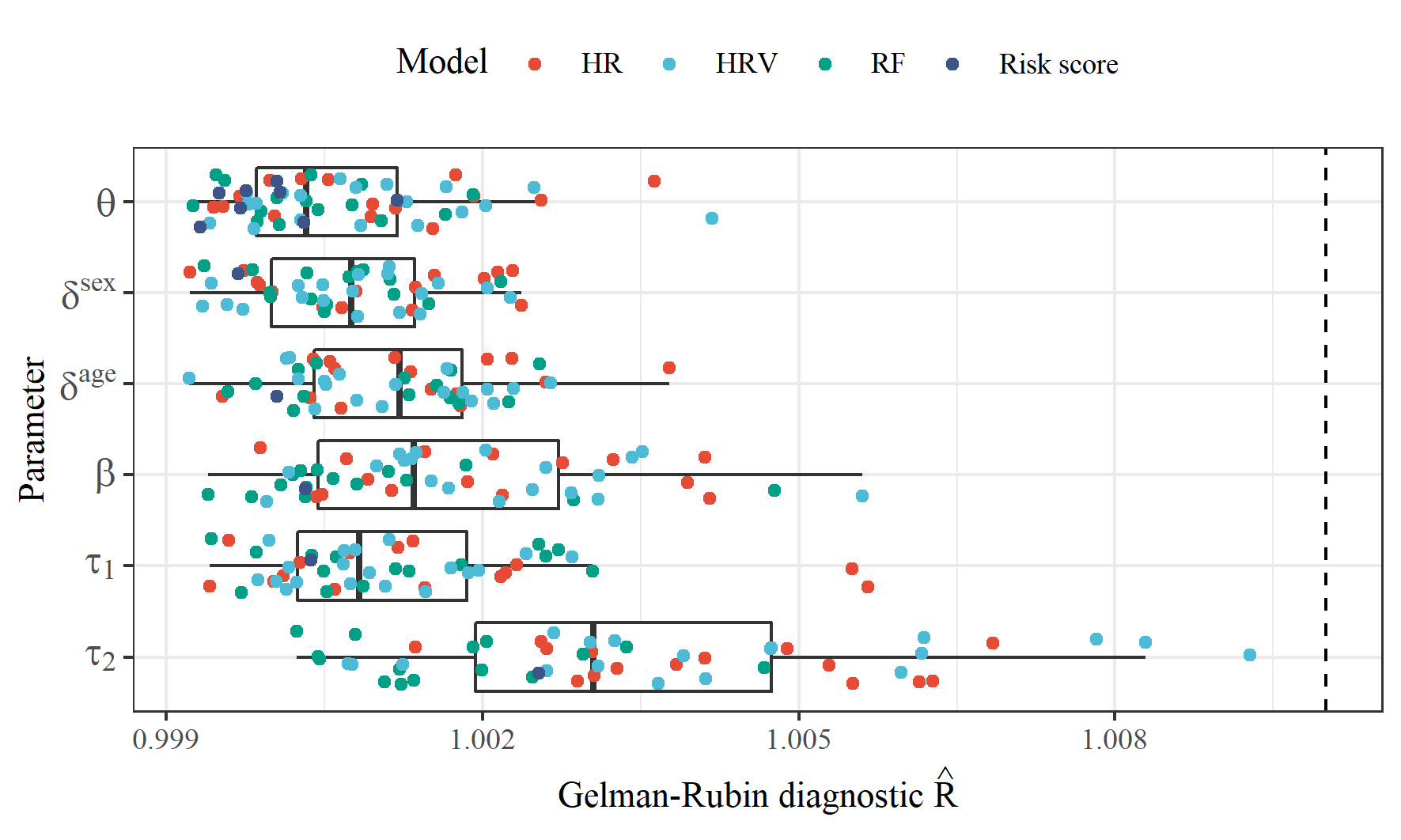

Figure 10: **Gelman-Rubin convergence diagnostic (**$\hat{\boldsymbol{R}}$**).** Shown are the $\hat{R}$ values (dots and boxplots) for parameters of the risk score model and the explanatory models. Values above 1.01 can be an indicator of convergence problems [1]. However, no values exceeded the critical threshold, thus indicating convergence of the chains.

## References

1. Gelman A, Rubin DB. Inference from Iterative Simulation Using Multiple Sequences. *Statistical Science*. 1992;7(4):457-472. doi:10.1214/ss/1177011136
