# Supplementary material for: A Scalable Risk-Scoring System Based on Consumer-Grade Wearables for Inpatients With COVID-19: Statistical Analysis and Model Development
Source: JMIR Form Res. 2022 Jun 21;6(6):e35717. doi: 10.2196/35717 (PMC9217156; doi:10.2196/35717)
Supplement: Multimedia Appendix 4 [file formative_v6i6e35717_app4.docx]

# Multimedia Appendix 4. Robustness of Explanatory Analysis.

## D Robustness of Explanatory Analysis

In our explanatory analysis, we estimated the relationship between physiological features and patient outcomes using Bayesian survival models as specified in Section “Explanatory analysis of the association of physiological features with patient outcomes”. Here, we assess the robustness of our estimates with regard to (i) the time window used for physiological measurements, (ii) the specification of the time trend, (iii) the modeling of subject-specific variation, (iv) the choice of the distribution function for the cumulative probability model, and (v) the main coefficient prior. For reasons of brevity, we report estimates for one feature each of HR, HRV, and RF in the following. The results are similar for all other features.

## D.1 Time Window

In our main analysis, we used measurements during the time window 0:00a.m. – 5:00a.m. The choice of this time window was motivated by the activity patterns of patients and the associated changes in physiological measurements throughout the day (i.e., during the time frame, there should be little distortion due to patient activity). Nevertheless, we wanted to check the sensitivity of our results with respect to different time windows. Therefore, we fitted models with a time window that was one hour shorter or longer than in the main analysis. The resulting estimates for mean HR, HRV RMSSD, and mean RF from each model are shown in Figure 10. As can be seen, our estimates are not sensitive to the particular choice of morning hours included. This thus adds to the robustness of our modeling.

In addition, we tested a time window of 24 hours length, i.e., from 5:00a.m. on the previous day until 5:00a.m. on the current day. The corresponding estimates for mean HR, HRV RMSSD, and mean RF are shown in Figure 10. We also computed a risk score using physiological features from the 24 hour time window and evaluated its performance using the same approach as in the main analysis. The risk score based on the 24 hour time window showed a favorable performance with an AUROC that was consistently above 0.5 for different time horizons between one and five days. However, the credible intervals were wide, thus reflecting larger variability in sensor measurements that span a longer time window. The latter supports our choice of a time window which only covers the phase of patients’ night rest, and further suggests that careful feature engineering is important to make reliable inferences from physiological features when predicting patient outcomes.


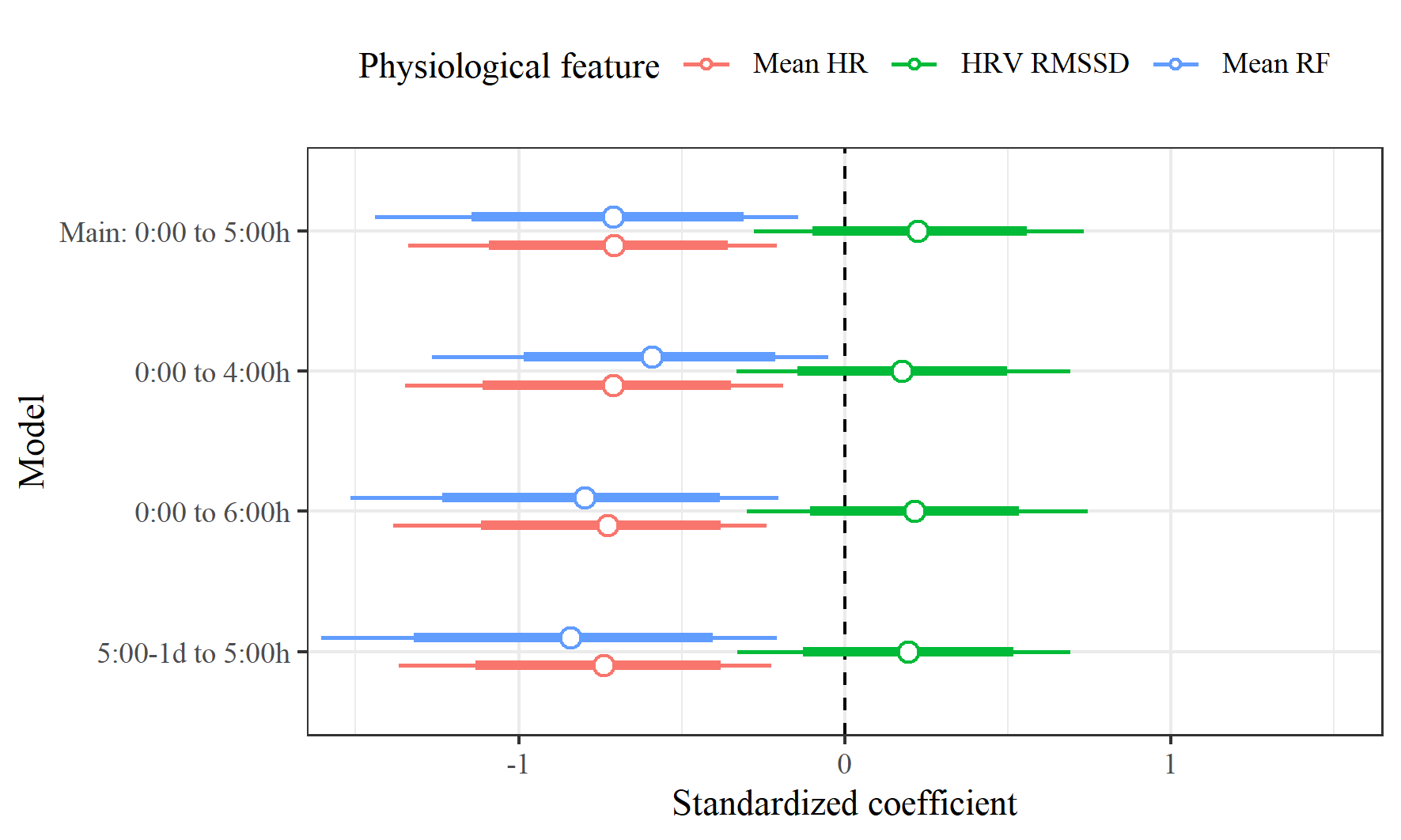
Figure 11: **Sensitivity of models to different time windows.** Shown are the standardized coefficients of physiological features from models using a time window from (i) 0:00a.m. – 5:00a.m. (main model), (ii) 0:00a.m. – 4:00a.m., (iii) 0:00a.m. – 6:00a.m, or (iv) 5:00a.m. – 5:00a.m (24h). Reported are posterior means (dots) and the 80% and 95% credible intervals (thick and thin bars).

### D.2 Alternative Specifications of Time Trend

In our study, we used survival models with a time trend component to account for changes in patient condition over time. In the main analysis, a linear time trend component was used. We here test alternative models with a different functional form for the time trend as part of our robustness checks. Specifically, we tested (i) one model with a quadratic time trend and (ii) one model where the time trend was modeled via a smoothing spline, i.e., a thin plate regression spline. The results as shown in Figure 11. Both models yielded estimates similar to the main model.


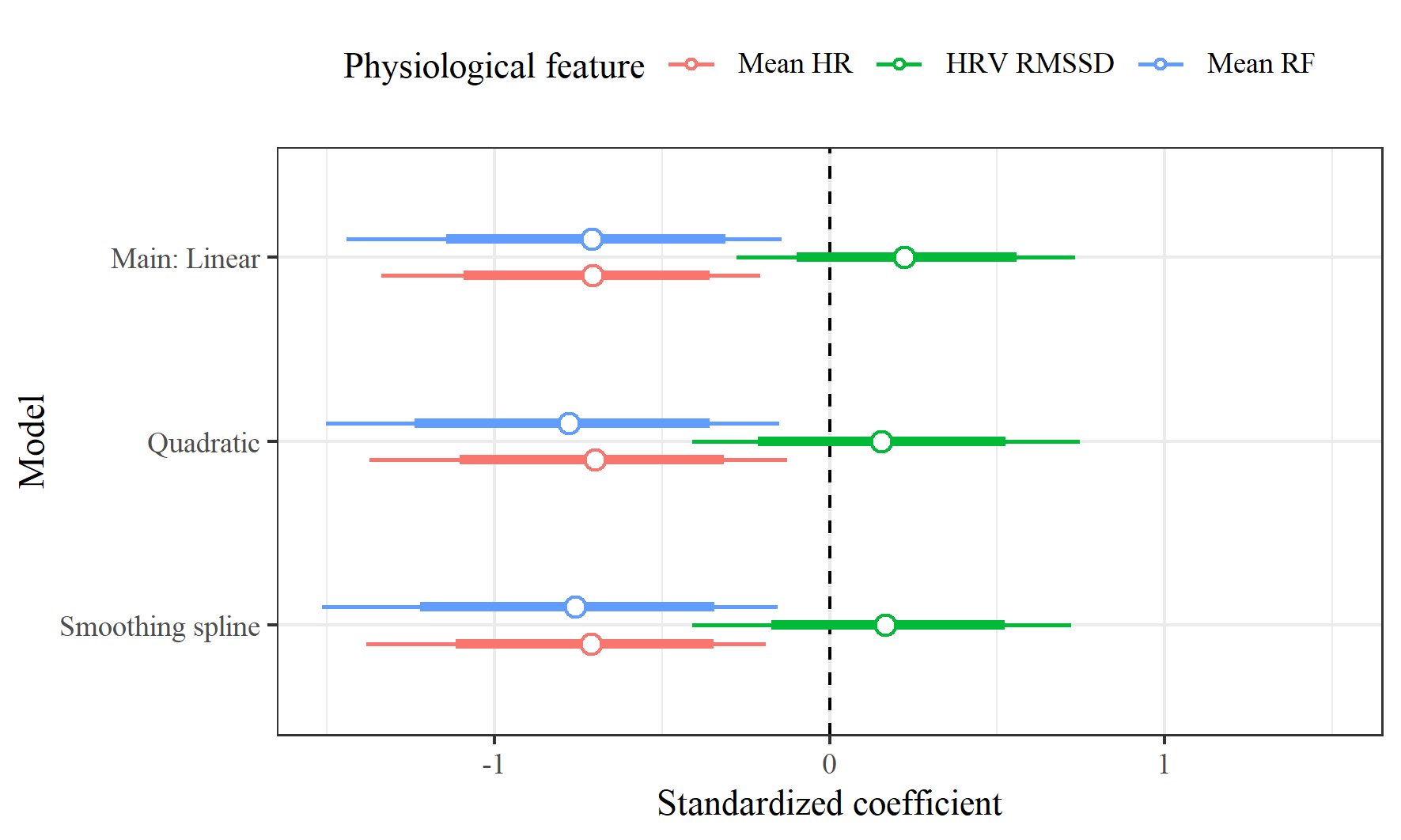


Figure 12: **Estimates from models with different time trend specifications.** Shown are the standardized coefficients of physiological features from models using (i) a linear (main model), (ii) a quadratic, and (iii) a time trend based on smoothing splines. Reported are posterior means (dots) and the 80% and 95% credible intervals (thick and thin bars).

### D.3 Subject-specific Variation

In our main paper, we modeled variation in the health condition between patients as follows: (i) Patient age and sex were included as predictors to adjust for potential differences in the health condition as well as potential differences in the physiological features. (ii) A random effect term was used to model further variation across patients not accounted for by age and sex. As a robustness check, we compare our main model with alternative models where we either exclude the demographic predictors, the random effect term, or both.

The results confirm those from the main paper (Figure 12). (i) The estimates for physiological features remain widely unchanged upon exclusion of the demographic predictors. (ii) The estimates become slightly smaller upon exclusion of the random effect term. The 80% credible intervals for each feature overlap across all models. These findings indicate low sensitivity of the results to the specific controls used but also suggest the presence of subject-specific variation in health condition not captured by patient age or sex. Therefore, the results remain robust when controlling for subject-specific variation.


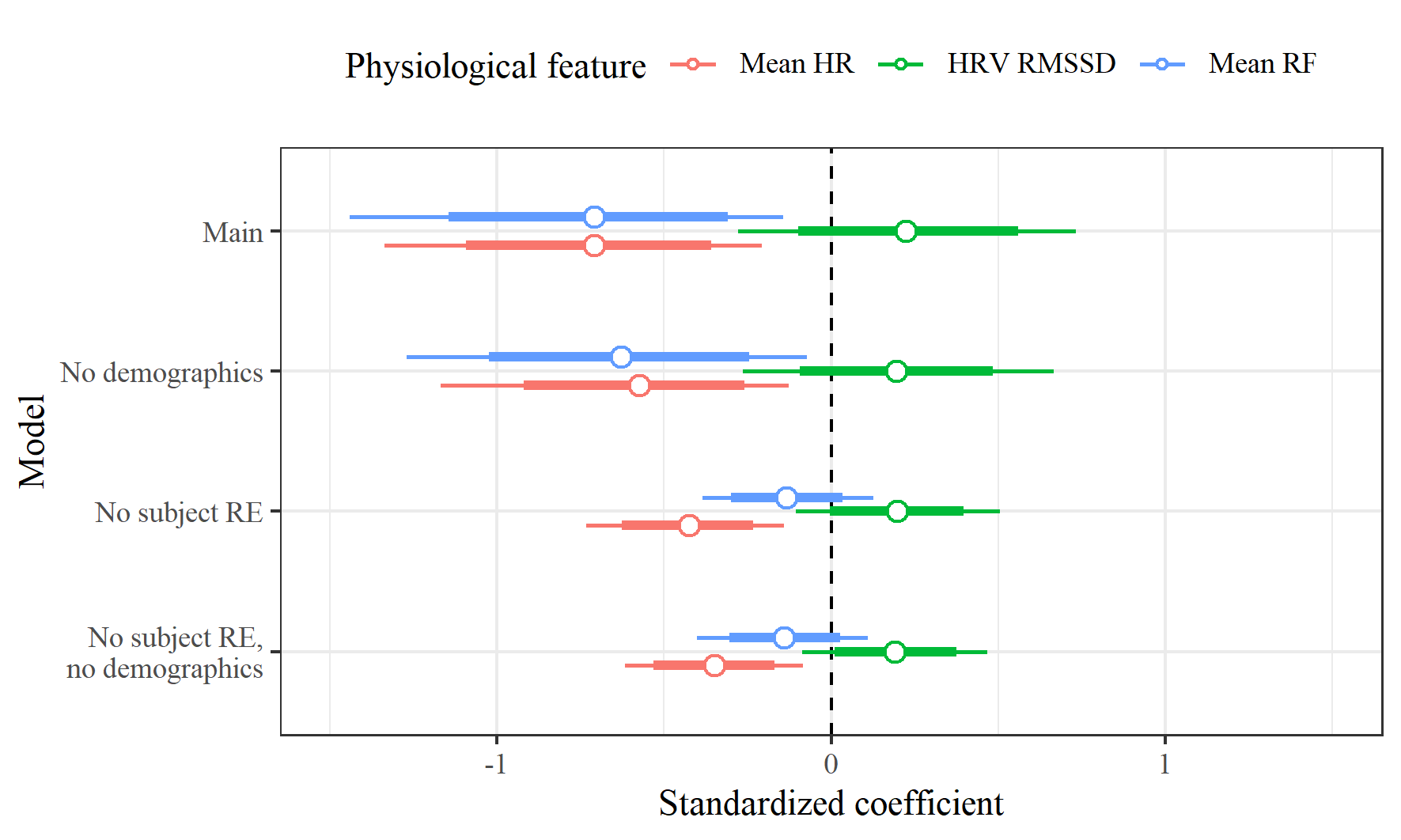


Figure 13: **Estimates from models with different controls for subject specific variation.** Shown are the standardized coefficients of physiological features from models (i) including both demographic predictors and a patient random effect (main model), (ii) excluding demographic predictors, (iii) excluding the patient random effect, and (iv) excluding both demographic predictors and the patient random effect, respectively. Reported are posterior means (dots) and the 80% and 95% credible intervals (thick and thin bars).

### D.4 Sensitivity to Distribution Function

We specified the distribution function of the cumulative probability model in the main analysis as an extreme value distribution, corresponding to the use of a complementary log-log link. This choice is theoretically justified since the present survival analysis is based on observations resulting from a grouping of continuous time into intervals [1]. Still, we also tested models with (i) a logistic distribution, corresponding to a logit link and (ii) a normal distribution, corresponding to a probit link. Figure 13 shows the resulting estimates for each model. The estimates from the model with a normal distribution have a slightly smaller uncertainty than those from models with an extreme value or logistic distribution. Across all models, the results are qualitatively similar.


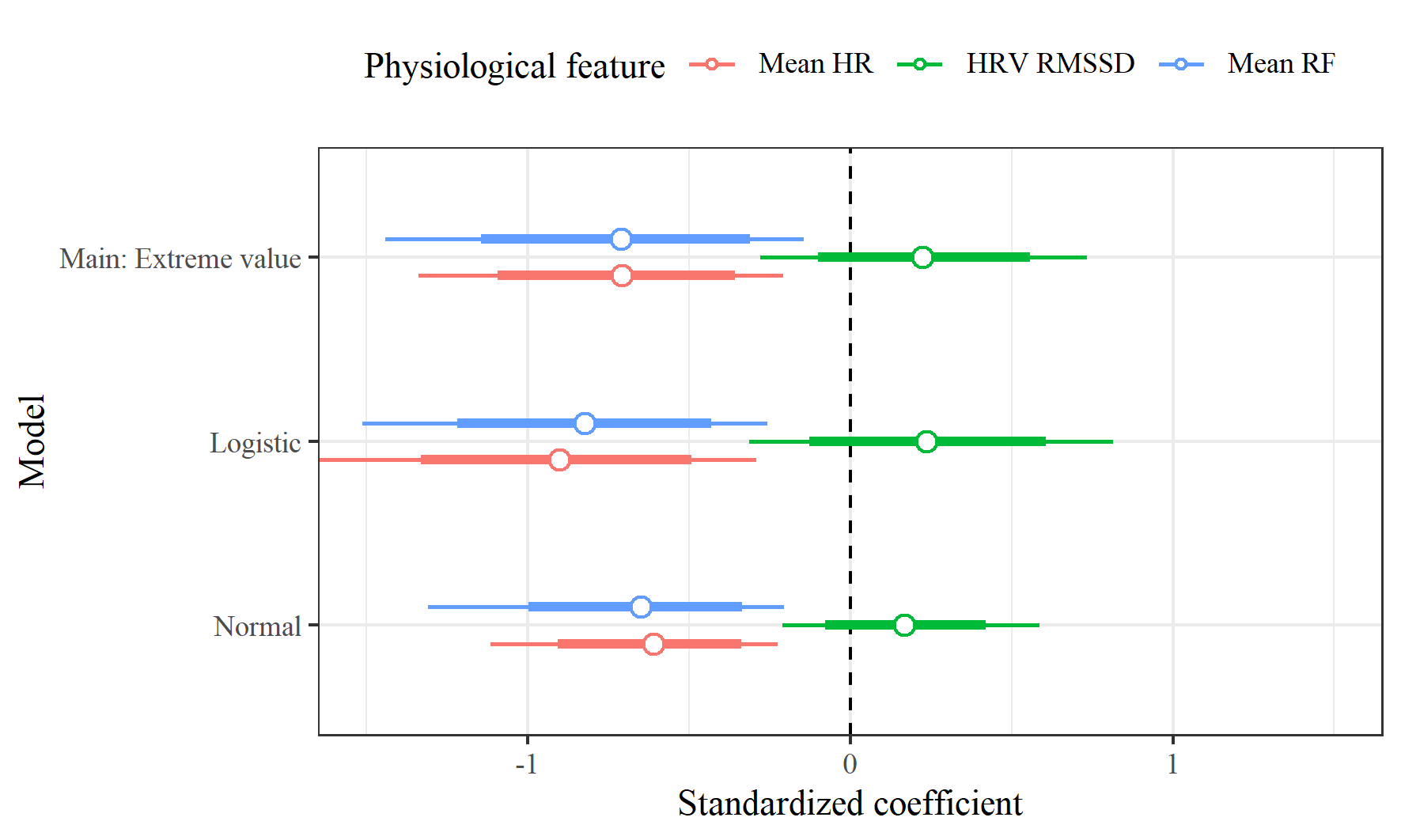


Figure 14: **Estimates from models with different distribution functions.** Shown are the standardized coefficients of physiological features from models using (i) an extreme value distribution (main model), (ii) a logistic distribution, and (iii) a normal distribution. Reported are posterior means (dots) and the 80% and 95% credible intervals (thick and thin bars).

### D.5 Sensitivity to Main Coefficient Prior

We chose weakly informative priors for all models and parameters. These priors are centered at zero, making them neutral with regard to the direction of the relationship between independent and dependent variables, and sufficiently wide to provide some regularization during sampling without having a relevant impact on parameter estimates. To further ensure that our results are not dependent on our priors, we conducted an additional robustness check in which we doubled the standard deviation of our main coefficient priors, making them much flatter than the original priors. This expresses larger uncertainty in the range of possible parameter values. Nevertheless, we obtain almost identical parameter estimates with the widened priors. This confirms that our results are informed by the data and not by our priors.


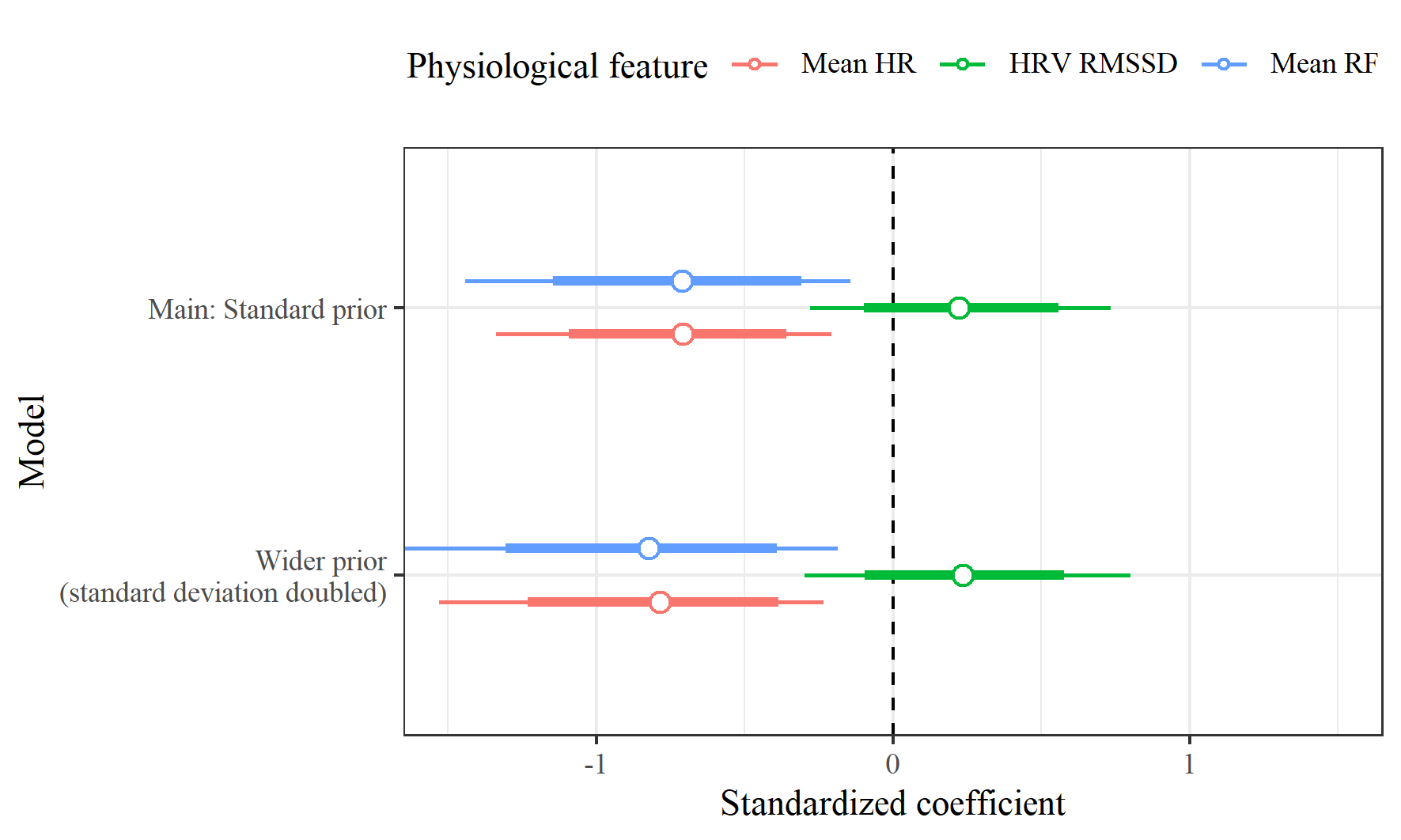
 Figure 15: **Estimates from models with different main effect priors.** Shown are the standardized coefficients of physiological features from models using (i) a standard normal prior (main model), and (ii) a normal prior with double standard deviation. Reported are posterior means (dots) and the 80% and 95% credible intervals (thick and thin bars).

## References

1. Kalbfleisch JD, Prentice RL. *The Statistical Analysis of Failure Time Data*. 2nd ed. John Wiley & Sons; 2002. doi:10.1002/9781118032985
