# Supplementary material for: A Scalable Risk-Scoring System Based on Consumer-Grade Wearables for Inpatients With COVID-19: Statistical Analysis and Model Development
Source: JMIR Form Res. 2022 Jun 21;6(6):e35717. doi: 10.2196/35717 (PMC9217156; doi:10.2196/35717)
Supplement: Multimedia Appendix 5 [file formative_v6i6e35717_app5.docx]

# Multimedia Appendix 5. Principal component analysis (PCA) results.

## E PCA Results


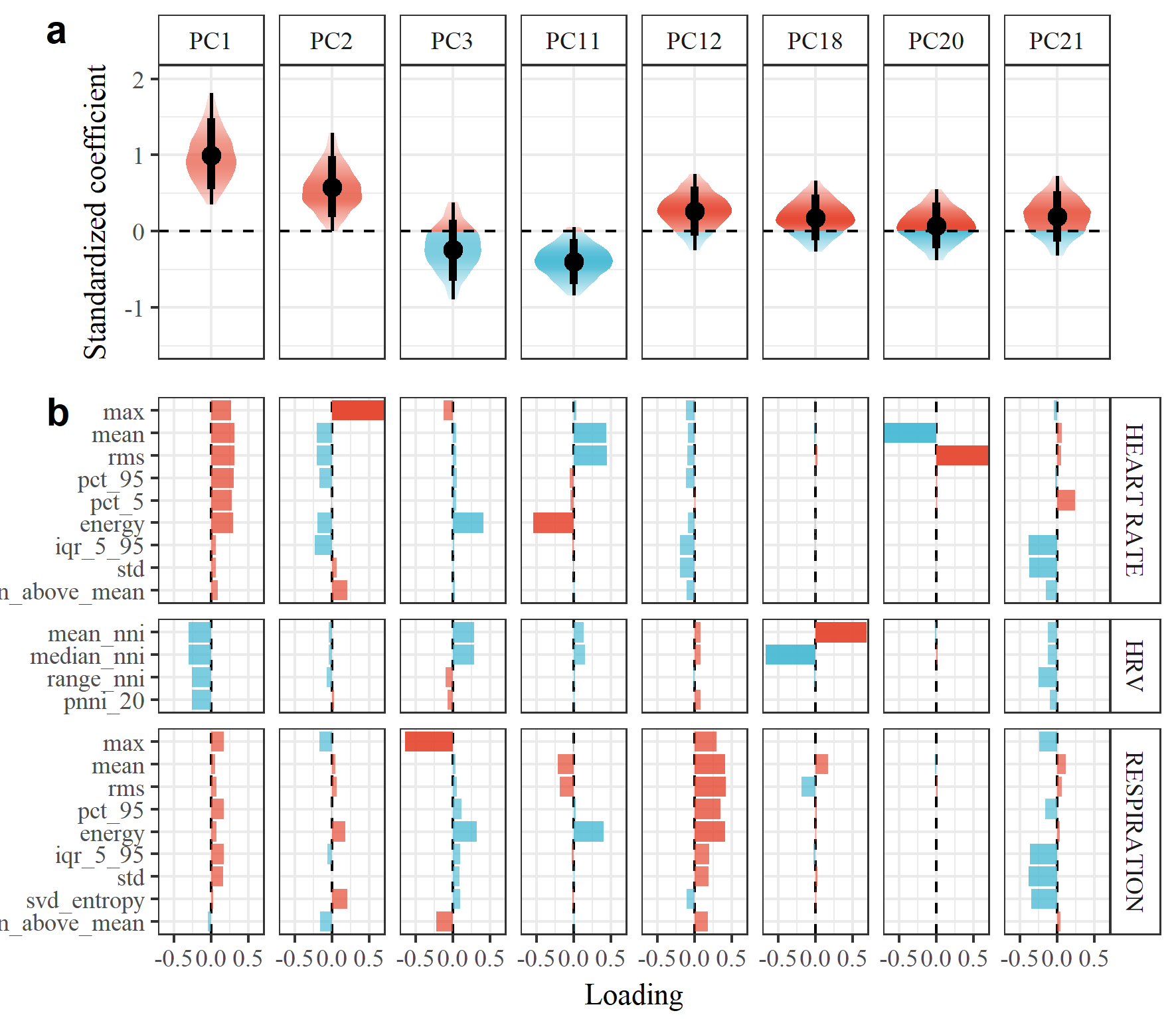


Figure 7. **Principal components combined into an overall risk score.** Eight principal components (PCs) were selected for the risk score through pooled logistic LASSO regression. (a) Standardized coefficients of the PCs. In each column, the standardized coefficient of one PC in the risk score is reported. Shown are the posterior probability mass with mean (dot) and the 80% and 95% credible intervals (thick and thin bars). Positive values (red) indicate an association with a deterioration in the health condition, and negative values (blue) indicate an association with an improved health condition. (b) Loadings of the PCs. In each column, the loadings of physiological features onto the respective PC are shown. Features with positive loading are positively associated with the PC, and features with negative loading are negatively associated with the PC. Colors indicate whether, through the respective PC, features are associated with a deterioration in the health condition (red) or an improved health condition (blue).
