# Supplementary material for: A Scalable Risk-Scoring System Based on Consumer-Grade Wearables for Inpatients With COVID-19: Statistical Analysis and Model Development
Source: JMIR Form Res. 2022 Jun 21;6(6):e35717. doi: 10.2196/35717 (PMC9217156; doi:10.2196/35717)
Supplement: Multimedia Appendix 6 [file formative_v6i6e35717_app6.docx]

# Multimedia Appendix 6. Comparison to a Risk Score Using Only Demographic Features.

## F Comparison to a Risk Score Using Only Demographic Features

To further assess the added value of the physiological features used in our risk score, we additionally compared the performance of our risk score to a “null model” risk score that used a patient random effect, time trend and demographic features (patient age and sex) as in our main model, but no further predictors.

The performance of this “null model” risk score was evaluated via leave-one-subject-out cross-validation, analogous to our main risk score. The cross-validation results for the risk score using only demographics show a time-dependent AUROC consistently and significantly below 0.5 for a length-of-stay between one and six days. This result indicates overfitting on the training data due to a lack of predictive value of the covariates, leading to a below “chance level” performance in out-of-sample prediction.

Overall, the additional comparison establishes that a risk score solely based on demographics has no predictive value with regard to the time-varying health condition of the patients in our sample. This in turn confirms the added value of the physiological features used in our risk score.


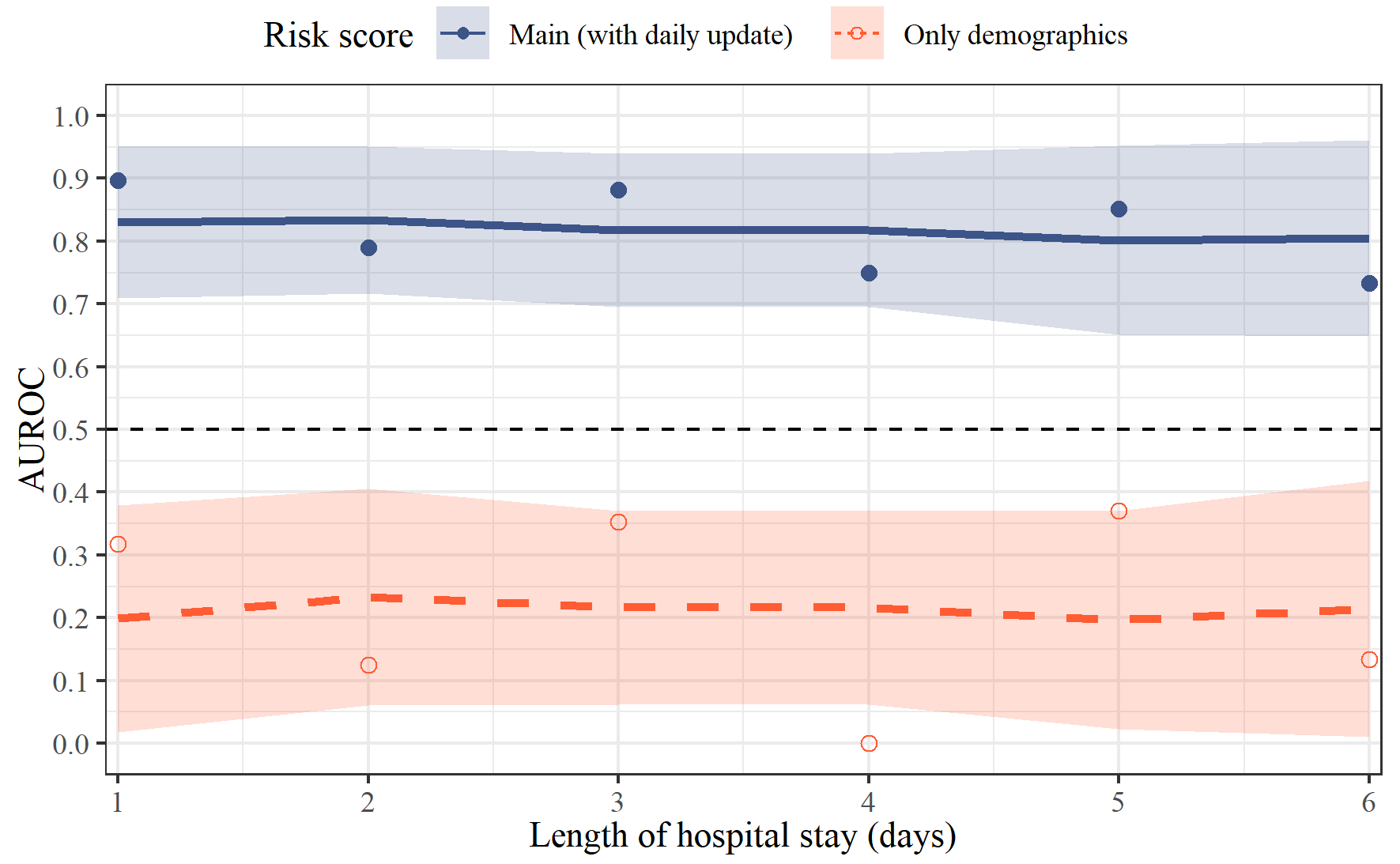


Figure 16: **Prediction performance of a risk score using only demographic features.** Shown is the time-dependent area under the receiver operating characteristic curve (AUROC) of the risk score in predicting patient discharge over time. The main risk score (blue, solid line) is compared with a risk score using only demographic features but no other predictors (red, dashed line). Out-of-sample predictions were obtained via leave-one-patient-out cross-validation. Dots show the individual time-dependent AUROC estimates for days with observed patient discharge. Smoothing was performed via a nearest neighbor estimator (see Performance Evaluation) to obtain an estimate of mean AUROC over time (lines) with 95% confidence intervals (shaded areas).
